# Regulation of social interaction in mice by a frontostriatal circuit modulated by established hierarchical relationships

**DOI:** 10.1038/s41467-023-37460-6

**Published:** 2023-04-29

**Authors:** Robert N. Fetcho, Baila S. Hall, David J. Estrin, Alexander P. Walsh, Peter J. Schuette, Jesse Kaminsky, Ashna Singh, Jacob Roshgodal, Charlotte C. Bavley, Viraj Nadkarni, Susan Antigua, Thu N. Huynh, Logan Grosenick, Camille Carthy, Lauren Komer, Avishek Adhikari, Francis S. Lee, Anjali M. Rajadhyaksha, Conor Liston

**Affiliations:** 1 Feil Family Brain and Mind Research Institute, Weill Cornell Medicine, New York, NY USA; 2 Weill Cornell/Rockefeller/Sloan Kettering Tri-Institutional MD-PhD Program, New York, NY USA; 3 Department of Pediatrics, Division of Pediatric Neurology, Weill Cornell Medicine, New York, NY USA; 4 Department of Psychology, University of California, Los Angeles, Los Angeles, CA USA; 5 Department of Psychiatry, Weill Cornell Medicine, New York, NY USA; 6 Weill Cornell Autism Research Program, New York, NY USA

**Keywords:** Neural circuits, Social behaviour, Stress and resilience

## Abstract

Social hierarchies exert a powerful influence on behavior, but the neurobiological mechanisms that detect and regulate hierarchical interactions are not well understood, especially at the level of neural circuits. Here, we use fiber photometry and chemogenetic tools to record and manipulate the activity of nucleus accumbens-projecting cells in the ventromedial prefrontal cortex (vmPFC-NAcSh) during tube test social competitions. We show that vmPFC-NAcSh projections signal learned hierarchical relationships, and are selectively recruited by subordinate mice when they initiate effortful social dominance behavior during encounters with a dominant competitor from an established hierarchy. After repeated bouts of social defeat stress, this circuit is preferentially activated during social interactions initiated by stress resilient individuals, and plays a necessary role in supporting social approach behavior in subordinated mice. These results define a necessary role for vmPFC-NAcSh cells in the adaptive regulation of social interaction behavior based on prior hierarchical interactions.

## Abstract (web summary)

Here, the authors identify a frontostriatal circuit that is involved in regulating social interactions based on learned hierarchical relationships.

## Introduction

Social hierarchy is a nearly ubiquitous aspect of life and an important regulator of behavior in many species, including humans, non-human primates, birds, fish, and rodents^1–8^. Hierarchical relationships emerge in part through repeated bouts of social competition, in which one animal establishes dominance over a subordinate competitor^9^. Once established, they are generally stable and exert a powerful influence on subsequent behavior^9–11^. Dominant animals exhibit increased social aggression, territorial defensiveness, and body mass while subordinate animals are typically smaller in size and submit more quickly to dominant behavioral displays^9,10^. Submitting to a dominant social partner may yield short-term benefits, such as avoiding injury; however, excessive social avoidance can also be maladaptive in the long term, for example by restricting access to prospective mates and other critical resources^12^. Thus, while submission can be a beneficial strategy, challenging dominant partners and initiating social approach behavior in social hierarchical contexts may also be important for long-term survival.

The neurobiological mechanisms that encode and regulate hierarchical interactions are not well defined, but converging data from multiple species indicate that the medial prefrontal cortex (PFC) may be involved^1,13–16^. In mice, social dominance has been associated with increased synaptic strength in the dorsomedial PFC, and neurons within this region encode the behavior of individuals and their social partners during social interactions^17,18^. In a separate study, dorsomedial PFC neurons exhibited increased firing rates during social competition, and optogenetic manipulation of dorsomedial PFC activity was sufficient to alter the outcome of these contests and subsequent measures of hierarchical status^11^. Although these studies did not evaluate other prefrontal areas, human neuroimaging data suggest that the ventromedial PFC may also be important, especially for perceiving social stimuli, assessing social hierarchy, and using that information to regulate behavior^2,19–22^. In accord with these observations, neurological patients with focal ventromedial PFC lesions exhibit prominent deficits in both evaluating social hierarchy and responding appropriately to social cues^23^. Furthermore, ventromedial PFC dysfunction is a common neurobiological correlate of multiple neuropsychiatric conditions featuring atypical social behavior^22,24–29^.

Still, how ventromedial PFC circuits may contribute to the perception of social hierarchy and the adaptive regulation of social interaction behavior remains unclear. Importantly, PFC neurons are functionally heterogeneous, and it is unknown whether particular neuronal subtypes support these functions through projections to specific downstream targets. However, several lines of evidence suggest that PFC projections to the nucleus accumbens (NAc) are a likely candidate. The infralimbic area of the ventromedial PFC projects predominantly to the shell subregion of the NAc (NAcSh)^30–32^. Human neuroimaging studies show that the ventromedial PFC is responsive to social rewards and social status, and theoretical models posit a role for the NAc in encoding expected value representations and optimizing reward-seeking behavior in both social and non-social contexts^2,33–35^. In animal models, the PFC and NAc have been repeatedly implicated in both reward-seeking behavior and in regulating social interactions, especially after chronic stress^15,16,36–42^. Interestingly, in at least one recent study, stimulating PFC projections to NAc was sufficient to increase social interaction behavior after chronic social defeat stress^43^. Although this study was not restricted to vmPFC neurons and did not directly examine the activity of vmPFC-NAcSh cells during social competitions or establish a specific role for this circuit in regulating *hierarchical* social interactions, the results lend further support to the hypothesis that the PFC-NAc circuit plays a critical role in evaluating social hierarchy and mediating its impact on social interaction behavior.

To test this hypothesis, we used fiber photometry and chemogenetic tools to record and experimentally manipulate the activity of NAcSh-projecting vmPFC neurons during social competition and during social interaction behavior after repeated defeats^44,45^. We found that vmPFC-NAcSh cells signal learned hierarchical relationships, modulating their activity state during social competition based on the known hierarchical status of the partner mouse. This projection is required for initiating effortful social dominance behaviors, specifically when a subordinate mouse encounters a dominant competitor within an established hierarchy. Furthermore, we found that after repeated bouts of social defeat, this circuit is preferentially engaged during social interaction in repeatedly subordinated, stress-resilient individuals (but not in stress susceptible or unstressed individuals) and is required for supporting social approach behavior. Together, these results suggest that the vmPFC-NAcSh circuit plays a specific role in regulating social interaction behavior by signaling social hierarchy information and mitigates social avoidance that is characteristic of both subordinate mice in established hierarchies and socially defeated mice.

## Results

### vmPFC-NAcSh activity is modulated by hierarchical status in social competitions

**Fig. 1:**
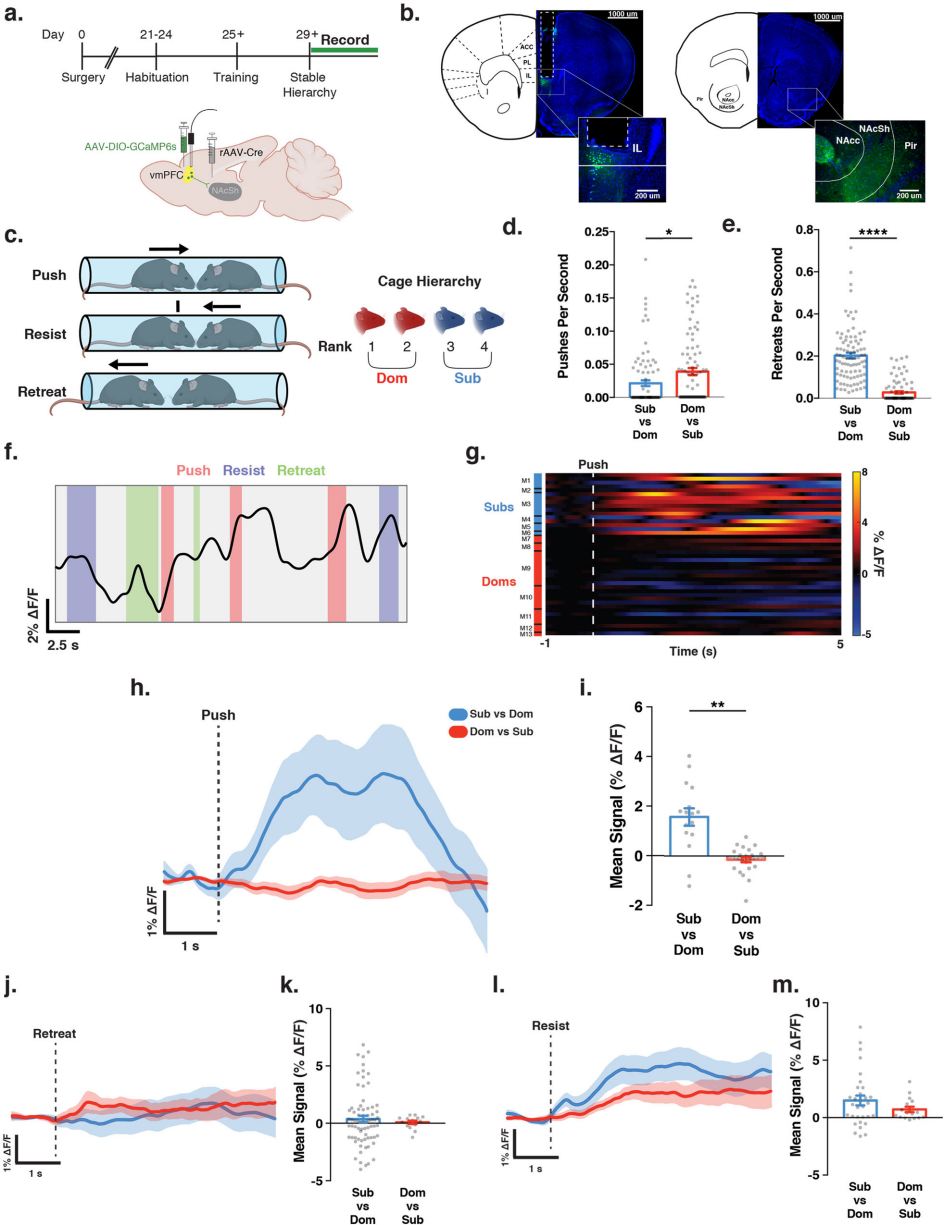
vmPFC-NAcSh activity is modulated by hierarchical status in social competition**s**. **a** Schematic of strategy for recording calcium activity from ventromedial prefrontal cortex cell bodies projecting to the nucleus accumbens shell (vmPFC-NAcSh) during tube test assay. Created with BioRender.com. **b** Representative images of fiber-optic tract and GCaMP6s expression in vmPFC-NAcSh cell bodies and terminals. Images modified from the Allen Reference Atlas—Mouse Brain^75^. **c** Schematic demonstrating tube test behaviors and classification of hierarchy based on within-cage rank. Created with BioRender.com. **d** Increased push rate in dominant mice against subordinate partners compared with subordinate mice against dominant partners. Welch’s unpaired *t* test, *T*_(173.3)_ = 2.46, *p < 0.015. (*N* = Sub vs Dom: 92 bouts, Dom vs Sub: 92 bouts, from 16 mice). **e** Increased retreat rate in subordinate mice against dominant partners compared with dominant mice against subordinate partners. Welch’s unpaired *t* test, *T*_(117.3)_ = 11.96, ****p < 0.0001. (*N* = Sub vs Dom: 90 bouts, Dom vs Sub: 91 bouts, from 16 mice). **f** Photometry trace of vmPFC-NAcSh activity during tube test assay. **g** Heatmap of vmPFC-NAcSh activity during all push events for all subordinate and dominant mice. **h** Mean vmPFC-NAcSh calcium activity centered on initiation of push separated by subordinate mice pushing a dominant competitor and dominant mice pushing a subordinate competitor. **i** Increased mean amplitude of vmPFC-NAcSh activity in subordinate mice pushing a dominant competitor vs dominant mice pushing a subordinate competitor. Linear mixed-effects model (LMM), *T*(42) = –2.98, **p = 0.005 for main effect of group on mean amplitude (*N* = subordinate group: 18 events from 6 mice; dominant group: 26 events from 7 mice). **j** Mean vmPFC-NAcSh calcium activity centered on initiation of retreat behavior separated by subordinate vs dominant or dominant vs subordinate bouts. **k** No group differences in vmPFC-NAcSh activity during retreat behaviors. LMM, *T*(88) = 0.235, *p* = 0.815 for main effect of group on mean amplitude (*N* = subordinate: 75 events from 6 mice; dominant: 15 events from 6 mice). **l** Mean vmPFC-NAcSh calcium activity centered on initiation of resist behavior separated by subordinate vs dominant or dominant vs subordinate bouts. **m** No group differences in vmPFC-NAcSh activity during resist behaviors. LMM, T(45) = −1.113, *p* = 0.272 (N = subordinate: 31 events from 6 mice; dominant: 16 events from 7 mice). All error bars equal mean ± SEM.

To investigate how the vmPFC-NAcSh circuit contributes to the perception of social hierarchy and the regulation of social interaction behavior, we used fiber photometry to record the activity of vmPFC cells projecting to NAcSh during social competition in the tube test, a commonly used and well-validated behavioral test for interrogating social hierarchies in mice^9,17,46^. To record neuronal activity in NAcSh-projecting vmPFC cells, we injected a retrograde-transported viral vector containing Cre-recombinase into NAcSh (rAAV2-retro-Cre^47^) and a Cre-inducible genetically encoded calcium sensor into vmPFC (AAV1-Syn-FLEX-GCaMP6s-WPRE). We then implanted an optical fiber over vmPFC to record from NAcSh-projecting vmPFC cells expressing the calcium sensor during tube test behavior (Fig. 1a, b; Supplementary Fig. 1). In the tube test assay, two mice are placed on opposite sides of a narrow open-ended tube, and each mouse attempts to exit the tube by pushing its partner and forcing it to retreat backwards toward the opposite end of the tube. Social hierarchy is evaluated within a given cage by testing each mouse against every other mouse in the cage, ranking them by number of wins and categorizing them as either “dominant” or “subordinate” within the cage (Fig. 1c). To ensure that the resulting assessments of social rank were reliable and stable, we repeated this round-robin evaluation process until individual ranks were consistent for four consecutive days of testing as per a standard protocol^46^ (Supplementary Fig 2a, b). In general, tube test assessments of social rank on Day 1 (after just one round-robin sequence of competitions) were strongly correlated with stable rankings defined over multiple days of testing (Supplementary Fig. 2c, d). To further validate these hierarchical categorizations, we used the urine marking assay, an established test of territorial dominance that is correlated with dominance in the tube test (see Methods)^10,48^. As expected, dominant mice marked a greater number of territories of larger size compared with subordinate animals, and the tube test and urine marking assay yielded consistent classifications of hierarchical status (Supplementary Fig. 2e, f) in line with previous work^17^.

After characterizing and validating stable hierarchical rankings as described above, we used fiber photometry to record neuronal activity in vmPFC-NAcSh cells in freely moving mice during tube test competitions with known cagemates. In order to accurately and objectively quantify specific behavioral events in the tube test, we used the DeepLabCut toolbox^49^ to train an algorithm to analyze video data and automatically identify three canonical tube test behaviors (see Methods, see Supplementary Fig. 3 for accuracy vs. hand-scored data): pushes (an effortful social dominance behavior), resists, and retreats (a subordinate behavior). In order to quantify the extent to which animals exhibited these behaviors while accounting for differences in the duration of individual tube test bouts, we measured the rate of each behavior (presented as behaviors per second). Similar to previous studies^11,17^, dominant animals competing with subordinate partners exhibited significantly more push behaviors (Fig. 1d) and fewer retreats (Fig. 1e; Supplementary Movies 1–3), relative to subordinate animals facing a dominant partner.

Next, we tested for changes in activity time-locked to the onset of each behavior (Fig. 1f; push, resist, and retreat). We found that during tube test competitions, vmPFC-NAcSh activity increased during effortful social dominance behavior, and this activity was modulated by the hierarchical status of the partner mouse. That is, there was a significant increase in vmPFC-NAcSh activity upon the initiation of push behaviors but only in a subordinate mouse facing a dominant partner (Fig. 1g–i). Supplementary analyses of activity in individual tube test bouts revealed that the magnitude of push-related vmPFC-NAcSh activity tended to scale with the difference in hierarchical status, and interestingly, vmPFC-NAcSh activity was most variable across individual encounters when the rank difference was small (Supplementary Fig. 4a). Importantly, push-related activity was observed only when individuals of a given rank encountered a more dominant partner but not when the same individuals encountered a more subordinate partner (Supplementary Fig. 4c). In contrast, there was no difference between subordinate and dominant mice in vmPFC-NAcSh activity during retreat (Fig. 1j, k, Supplementary Fig. 5a) or resist (Fig. 1l, m, Supplementary Fig. 5b) behaviors. These negative results support the specificity of this circuit-behavior relationship. We also found that differences in push-related vmPFC-NAcSh activity in subordinate vs. dominant animals were not attributable to differences in other characteristics of the push behavior (Supplementary Fig. 6a–c), including the duration of pushing and the likelihood of engaging in high-effort “body pushes” vs. lower-effort “nose pushes” (see Methods). Furthermore, we confirmed that vmPFC-NAcSh activity changes were not related to locomotion in the tube test or an open field arena (Supplementary Fig. 6d, e).

It is possible that vmPFC-NAcSh cells may send collateral projections to other downstream targets in addition to NAcSh. Thus, in order to both test the reproducibility of key findings and verify that activity recorded in vmPFC-NAcSh cell bodies was being transmitted to axon terminals specifically in NAcSh, we performed a second experiment in an independent cohort of mice, recording calcium signal from the terminals of the NAcSh-projecting vmPFC neurons during tube test behavior. To this end, we injected a viral vector (AAV1-hSyn-GCaMP6s) into the vmPFC and implanted the optical fiber over the lateral NAcSh (Supplementary Fig. 7a, b). Analyses of data from this independent cohort replicated all key findings in Fig. 1, including increased activity in vmPFC-NAcSh cells during push behavior in subordinate mice encountering a dominant partner (Supplementary Fig. 7c, d) but not during retreat or resist behaviors in either group of mice (Supplementary Fig. 7e–h). Together, these findings indicate that vmPFC-NAcSh cells are sensitive to hierarchical status during social competition, and increased activity in this projection neuron subtype is correlated with the initiation of effortful social dominance behavior selectively in subordinate individuals during encounters with a dominant competitor.

### vmPFC-NAcSh activity supports social dominance behavior in established hierarchies

**Fig. 2:**
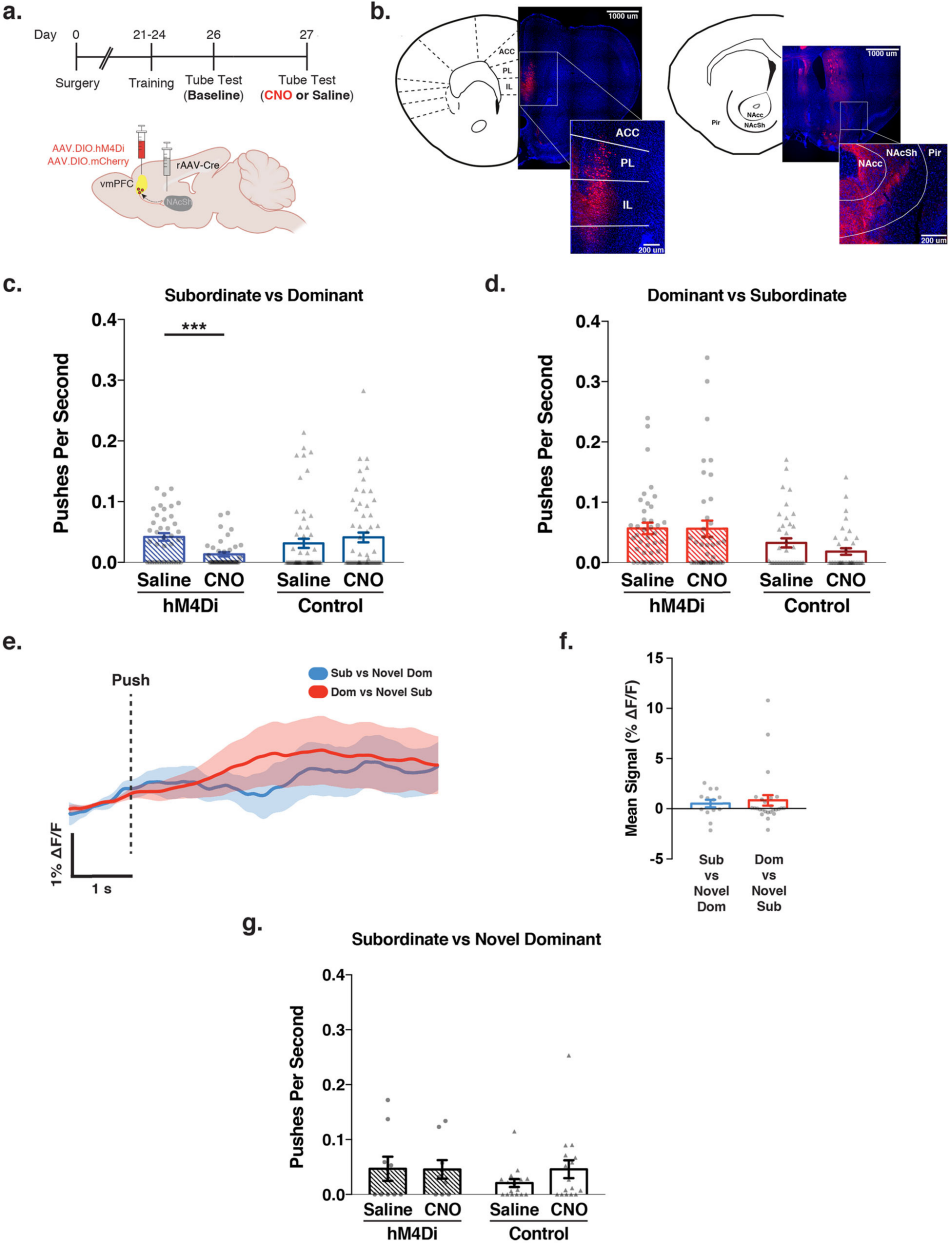
vmPFC-NAcSh circuit activity supports effortful social dominance behavior in previously established hierarchies. **a** Experimental tube test timeline and schematic of surgical strategy for viral injections for driving selective expression of hM4Di-mCherry inhibitory DREADDs or viral control in vmPFC-NAcSh cells. Created with BioRender.com. **b** Representative fluorescent images showing hM4Di-mCherry expression in vmPFC cell bodies and NAcSh terminals. *Abbreviations*: Anterior Cingulate Cortex (ACC), Prelimbic Cortex (PL), Infralimbic Cortex (IL), Nucleus Accumbens Core (NAcc), Nucleus Accumbens Shell (NAcSh), Piriform cortex (Pir). Images modified from the Allen Reference Atlas—Mouse Brain^75^. **c** Push rates for subordinate mice encountering a dominant competitor were significantly decreased after CNO injection in hM4Di-expressing mice but not in viral controls. Repeated measures two-way ANOVA (*N* = 40 hM4Di, 56 control; 2 experiments), Bonferroni corrected for post-hoc comparisons, significant interaction F(1,93) = 14.57, ***p < 0.001; CNO vs. saline in hM4Di group: *t*(98) = 3.666, ***p = 0.0003; CNO vs. saline in viral control group, *t*(98) = 1.546, *p* = 0.235). Data points indicate push rate for an individual trial. **d** Push rates for dominant mice encountering a subordinate competitor were not significantly altered after CNO injection in hM4Di-expressing mice or in viral controls. Repeated measures two-way ANOVA (*N* = 40 hM4Di, 40 control; 2 experiments), no interaction F(1,78) = 0.64, *p* = 0.423. Data points indicate push rate for an individual trial. **e** Mean (±SEM) photometry trace of vmPFC-NAcSh circuit activity time-locked to the initiation of pushes for subordinate mice encountering a novel dominant competitor (blue) and dominant mice encountering a novel subordinate competitor (red). **f** No group difference in vmPFC-NAcSh circuit activity associated with push behavior against novel partners. Linear mixed-effects model, T(37) = 0.383, *p* = 0.704 for main effect of experimental condition on mean amplitude (*N* = subordinate group: 13 push events from 5 mice—note 3 of 8 mice in cohort did not have any push events during their bouts with novel partners; dominant group: 26 push events from 8 mice). **g** Push rates for subordinate mice encountering a novel dominant competitor were not significantly altered after CNO injection in hM4Di-expressing mice or in viral controls. Repeated measures two-way ANOVA (*N* = 9 hM4Di, 16 control; 2 experiments), no interaction *F*(1,23) = 0.693, *p* = 0.414. Data points indicate push rate for an individual trial. All error bars presented as mean ± SEM.

The results above suggest that vmPFC-NAcSh activity may be required for promoting social dominance behavior in subordinate mice when they encounter a dominant partner. To test this hypothesis, we used a chemogenetic approach to suppress vmPFC-NAcSh activity in subordinate mice during tube test encounters with a dominant home cage competitor. We chose a chemogenetic manipulation in order to provide sustained inactivation of the vmPFC-NAcSh circuit across the duration of the tube test trials, in which naturalistic social dominance behaviors occur unpredictably, complicating efforts to deliver temporally specific optogenetic manipulations aligned to a certain behavior. To this end, we injected a retrograde-transported virus expressing Cre-recombinase (rAAV2-retro-Cre)^47^ into the NAcSh and a Cre-inducible Gi-DREADD (AAV8-hSyn-DIO-hM4Di-mCherry)^45,50^ into the vmPFC, yielding selective expression of Gi-DREADDs primarily in vmPFC-NAcSh cells (Fig. 2a, b; Supplementary Fig. 8a). Approximately three weeks later (allowing time for DREADD expression), we characterized the social hierarchy within each cage during a baseline day of behavior. As noted above, analysis of tube test training data from a separate cohort confirmed that cage hierarchies on the initial day of testing were strongly correlated with stable hierarchies following repeated testing (Supplementary Fig. 2b–d), supporting this approach. Thus, to avoid confounds from overtraining, our manipulation experiments were performed immediately following the initial baseline day of testing. We injected experimental and viral control subordinate mice with CNO and paired them in successive tube test trials with each saline-injected, dominant cage mate (Fig. 2a).

We found that suppressing vmPFC-NAcSh activity significantly reduced the rate of push initiations in subordinate mice during encounters with a dominant partner (Fig. 2c). In contrast, CNO had no effect on push initiations in subordinate mice expressing control virus (Fig. 2c), confirming that social competition behavior is stable over time, as previously reported^9,17^. Furthermore, the effect of inhibiting vmPFC-NAcSh activity was specific to push behaviors: suppressing vmPFC-NAcSh activity had no effect on winning, bout duration, retreat, or resist behaviors (Supplementary Fig. 8b–f). While suppressing vmPFC-NAcSh activity reduced the rate of push initiations in subordinate mice, the same manipulation had no effect on push initiations in dominant animals during encounters with a subordinate partner (Fig. 2d) or on any other behavior in the tube test (while controlling for behavior in the partner mouse; see Supplementary Fig. 8g–k). Inhibition of vmPFC-NAcSh also had no effect on overall locomotion in the open field test (Supplementary Fig. 8l).

In separate experiments, we also tested whether activation of vmPFC-NAcSh was sufficient to increase push behavior. We found that neither sustained chemogenetic nor high-frequency optogenetic activation of vmPFC-NAcSh cells altered push initiations (Supplementary Fig. 9a–f). To further inform our interpretation of this unexpected result and provide a positive control, we replicated the work of Zhou and colleagues^11^, which showed that optogenetic activation of dorsomedial PFC (dmPFC) neurons was sufficient to increase social dominance behavior in the tube test. In agreement with this prior study^11^, we found that optogenetic activation of dmPFC neurons significantly increased push rates and tended to drive increases in social rank in low-rank (subordinate) individuals (Supplementary Fig. 9g-j). Together, these results suggest that activity in both dmPFC neurons and vmPFC-NAcSh projections is required for supporting social dominance behavior, but that artificial stimulation of the vmPFC-NAcSh projection is not sufficient to drive increased push behavior. Together with the findings in Fig. 1, our data indicate that vmPFC-NAcSh activity is sensitive to the hierarchical status of a social competitor, and is required for initiating effortful social dominance behaviors, selectively in subordinate mice during encounters with a dominant competitor.

Previous studies suggest that prefrontal cortical circuits are important for modulating behavior by storing and retrieving various forms of memory^51–54^. This led us to hypothesize that vmPFC-NAcSh activity might regulate behavior during social competition specifically based upon previously learned information about a given social hierarchy. To test whether the effects above were specific to established social hierarchies like those occurring in long-term cagemates, we repeated the same photometry and chemogenetic inhibition experiments, but this time we paired mice with unfamiliar partners of opposing rank from another cage. Remarkably, when facing novel partners, vmPFC-NAcSh activity did not increase during push initiation and did not differ between subordinates and dominant animals (Fig. 2e, f), nor did it differ in any other behaviors (Supplementary Fig. 10). Furthermore, in contrast to the results observed in Fig. 2c, chemogenetic inhibition of vmPFC-NAcSh activity had no effect on push initiations in subordinate mice encountering novel, unfamiliar but dominant partners (Fig. 2g), nor did it significantly affect any other tube test behavior in these bouts in secondary exploratory analyses (Supplementary Fig. 11). These data indicate that vmPFC-NAcSh activity supports effortful social dominance behaviors only in subordinate mice competing with a dominant partner and only in the context of an established, previously learned hierarchy.

### Activation of vmPFC-NAcSh cells during social approach in stress resilient mice

By playing a necessary role in promoting the interaction of subordinate mice with known, socially dominant individuals based on past experience, we reasoned that this circuit may also be important for promoting social interaction with established dominant mice in other contexts such as following repeated social defeat. The chronic social defeat stress paradigm consists of repeated exposure to a highly polarized hierarchical dominance relationship and is a well-validated model of stress-induced social dysfunction and depression-related behavior, reliably eliciting social avoidance, anhedonia and anxiety-like behaviors in defeated mice^55^. This paradigm has also been used to investigate the neurobiological basis of stress susceptibility, as ~60% of mice exhibit a “susceptible” behavioral response involving social withdrawal, whereas ~40% of mice are classified as “resilient” and continue to interact socially despite their previous experience with repeated defeats^56,57^. Previous work has demonstrated that optogenetic stimulation of PFC projections to the accumbens increases social interaction behavior after chronic social defeat stress^43^. Previous studies were not designed to record or inhibit vmPFC-NAcSh activity during social behavior, but this important result raises the interesting but as yet untested possibility that the vmPFC-NAcSh circuit may function differently in stress resilient individuals and may facilitate stress resilient social approach behavior by overriding a tendency for social avoidance after repeated defeats.

**Fig. 3:**
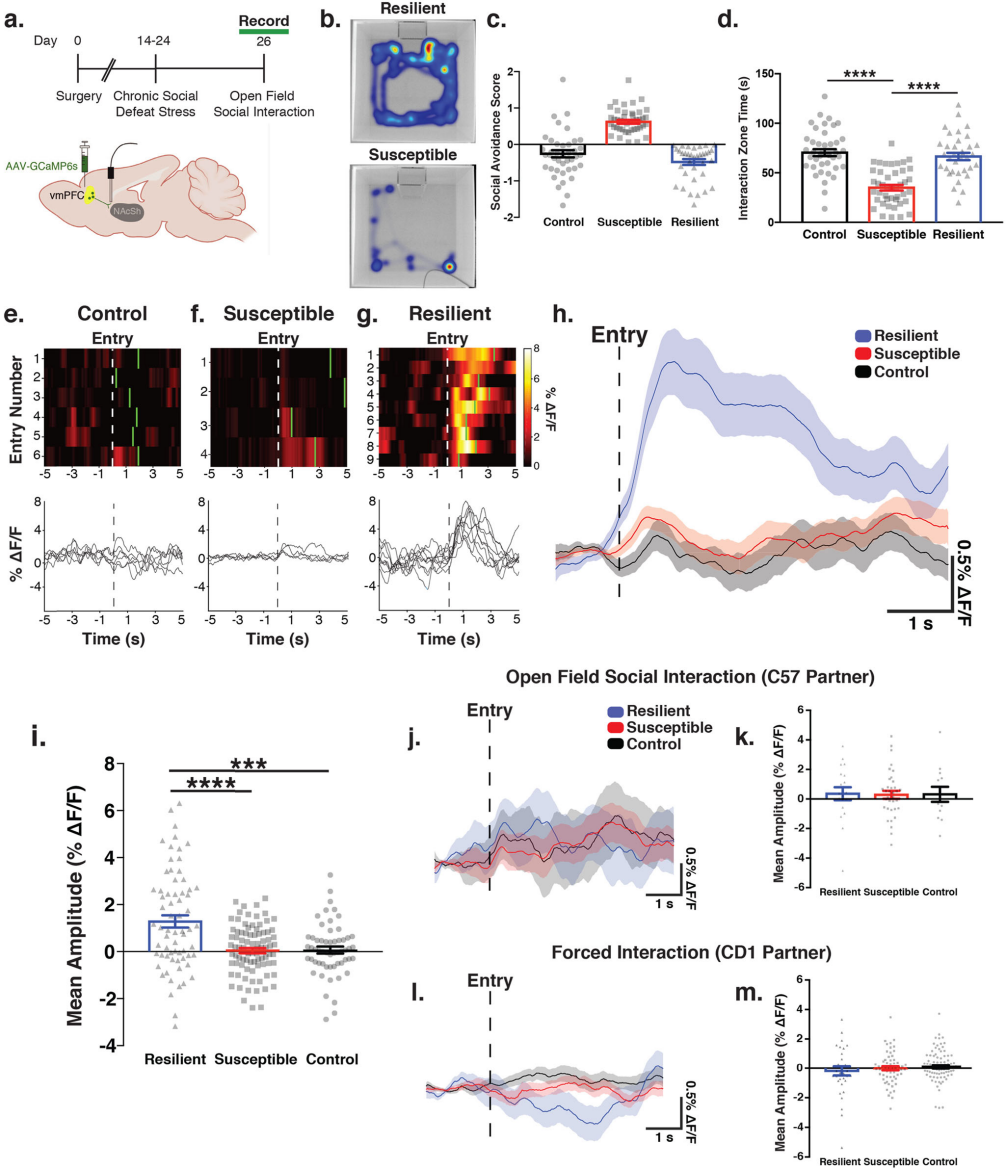
Selective activation of vmPFC-NAcSh cells during social approach in stress resilient mice. **a** Experimental strategy for recording vmPFC-NAcSh terminal activity during open field social interaction (OFSI). Created with BioRender.com. **b** Representative heatmaps of resilient and susceptible mice during OFSI. **c** Classification of animals using a social avoidance score (*N* = 119 animals, 8 experiments). **d** Stress-susceptible mice spent less time in the social interaction zone compared to other groups. One-way ANOVA (*N* = 119 animals, 8 experiments), Bonferroni corrected, F(2,116) = 36.08, ****p < 0.0001. Control vs. susceptible *t*(116) = 7.79, ****p < 0.0001, control vs. resilient, *t*(116) = 0.82, *p* > 0.99, susceptible vs. resilient, *t*(116) = 6.64, ****p < 0.0001. **e** Heatmap and photometry traces of vmPFC-NAcSh activity in each group during OFSI, each row shows an individual entry into the social interaction zone. Vertical green bars indicate mouse leaving the interaction zone. **f** As in **e**, but for a stress susceptible individual. **g** As in **e**, but for a stress resilient individual. **h** Photometry trace of vmPFC-NAcSh activity time-locked to interaction zone entry averaged across all entries and individuals in a given group. **i** Increased mean amplitude of vmPFC-NAcSh activity in the stress resilient group compared to the stress susceptible and unstressed control groups. Linear mixed-effects model (LMM) (*N* = group (entries, mice)–resilient (67,10), susceptible (86,19), control (57,13), 5 experiments), resilient vs. control: t(207) = 3.58, ***p = 0.0004; susceptible vs. control: t(207) = 0.11, *p* = 0.91; resilient vs susceptible t(207) = 3.80, ****p < 0.0001. **j** Photometry trace of vmPFC-NAcSh activity time-locked to interaction zone entry, but with a novel C57 interaction partner. **k** Mean amplitude of vmPFC-NAcSh activity during interactions with a novel C57 partner. LMM (*N* = group (entries, mice)–resilient (19,4), susceptible (35, 10), unstressed control (13,3); 1 experiment), resilient vs. control: t(64) = 0.067, *p* = 0.947; susceptible vs. control t(64) = –0.057, *p* = 0.955; resilient vs. susceptible: *t*(64) = 0.15, *p* = 0.882. **l** Photometry trace of vmPFC-NAcSh activity time-locked to interaction zone entry during forced interactions initiated by the CD1 partner mouse. **m** Mean amplitude of vmPFC-NAcSh activity during forced interactions initiated by the CD1 partner mouse. LMM (*N* = group (entries, mice)–resilient (31,3), susceptible (60,6), unstressed control (86,8), 1 experiment), resilient vs. control: t(174) = 1.78, *p* = 0.077; susceptible vs. control *t*(174) = –0.94, *p* = 0.35; resilient vs. susceptible t(174) = 0.97, *p* = 0.33. All error bars presented as mean ± SEM.

To address these questions, we tested for differences in vmPFC-NAcSh activity during social interaction behavior in repeatedly subordinated mice exposed to chronic social defeat stress, compared to unstressed control mice. We again used fiber photometry to record from the terminals of NAcSh-projecting vmPFC neurons. We recorded from these vmPFC-NAcSh projections during social interaction behavior after a standard ten-day social defeat stress paradigm, in which mice were exposed to repeated bouts of social defeat by a larger, aggressive, socially dominant CD1 mouse (Fig. 3a; see Methods). As per standard protocols^40,55,58^, mice were then tested in a 2-trial open field social interaction task, in which they freely explored an arena containing an empty perforated chamber (Trial 1) or a novel CD1 mouse confined to the chamber (Trial 2), and social interaction behavior was assessed in terms of time spent in an interaction zone that closely surrounds the chamber. As in prior work^59^, individual mice were classified as “stress resilient” or “stress susceptible” by calculating a social avoidance score, a Z-score based on the ratio of time each mouse spent in the interaction zone compared to the corner zones in the two trials of the task (see Methods). A high social avoidance score indicates that the test mouse tended to avoid the social contact zone when a CD1 partner mouse was present, as demonstrated in heatmaps of behavior from representative resilient and susceptible mice (Fig. 3b). As expected, susceptible mice had higher social avoidance scores (Fig. 3c), spent significantly less time in the interaction zone (Fig. 3d) and more time in the corner zones (Supplementary Fig. 12a) when the CD1 partner mouse was present, compared to both resilient mice and unstressed controls (Fig. 3c, d). There were no differences between groups in total distance traveled or the number of entries into the interaction zone (Supplementary Fig. 12b, c), indicating that differences in social avoidance or photometry signal cannot be attributed to non-specific changes in exploratory behavior. Furthermore, in a separate cohort of mice tested in an elevated plus maze and sucrose preference assay before and after chronic defeat stress, we found that differences in social avoidance in stress-susceptible vs. stress-resilient subgroups were specific to social interaction and were not associated with changes in anxiety-related behavior in the elevated plus maze or sucrose preference, which were equally affected by defeat stress in both subgroups (Supplementary Fig. 12d, e).

Next, we tested for changes in vmPFC-NAcSh activity time-locked to entry into the social interaction zone surrounding the CD1 partner mouse. We found that in stress resilient mice, vmPFC-NAcSh activity increased upon approach and entry into the interaction zone and tended to remain elevated for the duration of the social interaction (Fig. 3e–h; Supplementary Fig. 13). We also observed a statistically significant but much smaller increase in vmPFC-NAcSh activity in stress susceptible animals upon interaction zone entry (Fig. 3h, Supplementary Fig. 14a). However, vmPFC-NAcSh activity was significantly elevated during social interactions in stress resilient mice compared to stress susceptible mice as well as unstressed controls (Fig. 3i). To further understand the relationship between vmPFC-NAcSh activity and individual differences in social interaction behavior, we tested for correlations between activity and social avoidance score across individuals. Social avoidance score and vmPFC-NAcSh activity were significantly correlated within our stressed cohort but not in unstressed controls (Supplementary Fig. 14b, c). Importantly, there was no change in activity during interaction zone entries in unstressed control mice that had no prior experience with CD1s (Fig. 3e, h, i Supplementary Fig. 14a), despite the fact that their social interaction behavior was similar to the stress resilient group and consistent with our prior observations that activity in this circuit is sensitive to previous social interaction experiences.

These data show that vmPFC-NAcSh cells are selectively activated during social approach behavior in mice exposed to repeated bouts of defeat but not in unstressed control mice, suggesting (as in Figs. 1 and 2) that the functional role of this circuit is modulated by prior social dominance interactions. To further investigate this, we tested the same animals again in the open field social interaction task, but this time we substituted a novel, unfamiliar C57/BL6J partner mouse (instead of the standard CD1 partners that closely resemble the CD1 aggressors involved in repeated bouts of defeat). In contrast to the results in Fig. 3h, i, there were no significant differences in vmPFC-NAcSh activity during social interaction zone entry in the stress resilient, stress susceptible, or unstressed control groups (Fig. 3j, k; Supplementary Fig. 15a).

Also in agreement with our tube test data in Fig. 1, we found that the vmPFC-NAcSh circuit was activated only during social contacts initiated by the test mouse. To investigate this, we tested defeated mice and unstressed control mice in a forced interaction task, in which the experimental mouse was confined to a small center chamber, while a novel CD1 interaction partner freely explored the arena (see Methods). We tested for changes in vmPFC-NAcSh circuit activity time-locked to the CD1 partner mouse’s entry into the social contact zone. Unlike interactions initiated by the test mouse (Fig. 3h), interactions initiated by the CD1 partner mouse were not associated with significant changes in vmPFC-NAcSh activity in any of the three groups (Fig. 3l, m; Supplementary Fig. 15b), in accord with our findings in the tube test. Building on previously published optogenetic studies^43^, these fiber photometry experiments indicate that vmPFC-NAcSh functional activity is shaped by past social interaction experiences and may play an important role in determining adaptive, stress resilient behavioral responses: it is selectively active during social approach behavior in mice that were previously exposed to repeated defeats but retain a capacity for social engagement.

### vmPFC-NAcSh activity supports social approach behavior following repeated defeats

**Fig. 4:**
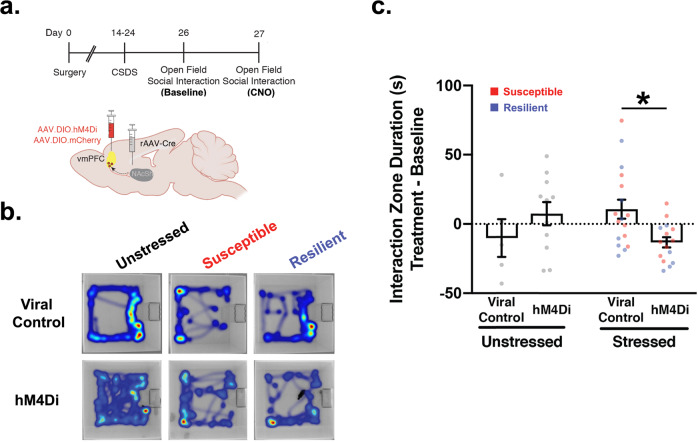
vmPFC-NAcSh activity supports social approach behavior following social defeat. **a** Experimental timeline and schematic of surgical strategy for viral injections for driving selective expression of hM4Di-mCherry inhibitory DREADDs or viral control in vmPFC-NAcSh cells. Created with BioRender.com **b** Heatmaps showing representative behavior of hM4Di-mCherry and viral control injected animals (unstressed controls, susceptible and resilient animals) after CNO injection. **c** Social interaction behavior (total interaction zone time) of stressed (susceptible (red) and resilient (blue)) hM4Di-expressing animals but not unstressed controls was significantly reduced by CNO injection but not viral control animals. Repeated measures two-way ANOVA (*N* = Unstressed: 5 viral control, 11 hM4Di; Stressed: 17 viral control, 15 hM4Di; 2 experiments), Bonferroni corrected for post-hoc comparisons, significant interaction F(1,44) = 6.71, *p = 0.013; Stressed viral control vs. stressed hM4Di: *t*(44) = 2.716, *p = 0.019; Unstressed viral control vs. unstressed hM4Di, *t*(44) = 1.317, *p* = 0.39). Error bars presented as mean ± SEM.

Finally, to test whether vmPFC-NAcSh circuit activity is required for supporting social approach behavior following chronic social defeat stress, we used chemogenetic tools to inhibit activity in this circuit during the open field social interaction task (see Methods) (Fig. 4a). As in Fig. 2, we injected a retrograde-transported virus expressing Cre-recombinase (rAAV2-retro-Cre)^47^ into the NAcSh and a Cre-inducible Gi-DREADD (AAV8-hSyn-DIO-hM4Di-mCherry)^45,50^ into the vmPFC, yielding selective expression of Gi-DREADDs in vmPFC-NAcSh cells (Supplementary Fig. 8). Mice were then exposed to a standard ten-day social defeat stress paradigm, followed by two days of open field social interaction testing (OFSI). Our data indicate that social avoidance scores remain relatively stable with repeat OFSI testing (Supplementary Fig. 16), justifying this approach to identifying susceptible vs. resilient individuals on the first day of OFSI testing and then manipulating vmPFC-NAcSh activity the following day. On the second day of OFSI testing, all mice were injected with CNO (see Methods).

We found that inhibiting vmPFC-NAcSh activity had different effects on chronically stressed mice compared to unstressed controls, significantly attenuating social interaction only in stressed mice previously exposed to repeated bouts of social defeat (Fig. 4b, c). In a similar control experiment where mice were injected with saline instead of CNO, this effect was not observed (Supplementary Fig. 17). Interestingly, although our experiment was not powered to detect distinct effects in stress-susceptible and stress-resilient individuals, the effects of chemogenetic inhibition were comparable in these two subgroups (Supplementary Fig. 18, see Discussion below). Together, these results indicate that vmPFC-NAcSh circuit activity is not only recruited during stress resilient behavior, but it is also required for supporting social approach behavior after repeated bouts of social defeat.

## Discussion

The neurobiological mechanisms that represent social hierarchies and mediate their influence on behavior are not well defined. Previous studies have shown that other areas of the medial PFC are modulated by social competitions and influence the outcome of social contests^11,13,14,17^, but relatively little is known about the role of ventromedial prefrontal areas or their projection targets. Our results indicate that a specific frontostriatal circuit projecting from the vmPFC to the nucleus accumbens shell (vmPFC-NAcSh) plays at least three distinct but related roles in this domain. First, we found that during social contests, vmPFC-NAcSh activity is modulated by the hierarchical status of a social competitor, such that activity is significantly greater when a mouse initiates an effortful social dominance behavior against a more dominant partner, compared to a more subordinate one. These results complement recent studies of the dorsomedial PFC, which showed that social competitions modify synaptic strength in this area^17^ and that dorsomedial PFC neurons signal the behavior of individuals and their social partners in a manner that is sensitive to their relative hierarchical status^18^. Our results show that ventromedial PFC neurons also signal learned social hierarchies and modulate hierarchical social interactions through projections to downstream targets in the NAc shell. It is worth noting that our results do not exclude the possibility that the vmPFC-NAcSh circuit is critical not only for encoding social hierarchy and adaptively regulating social approach behaviors, but also for exploiting other types of (non-social) rewards. Frontostriatal circuits regulate other forms of reward-seeking behavior by responding to rewarding and aversive stimuli, encoding reward-predictive cues, and processing valence^60–63^, and at least one study has shown that experimentally manipulating synaptic strength in infralimbic projections to NAc was sufficient to alter reward-related decision making^64^.

Second, we confirmed that the vmPFC-NAcSh circuit is required for initiating effortful social dominance challenges (push initiations) in a subordinate mouse competing with a dominant partner, but only in the context of an established hierarchy. In contrast, when a subordinate member of one cage hierarchy encounters a novel, unfamiliar, but socially dominant member of another cage hierarchy, inhibiting vmPFC-NAcSh cells had no effect on behavior. Together, these findings underscore the functional specificity of the circuit: it is selectively engaged during effortful social challenge behaviors (pushes, but not resist or retreat behaviors) but only when the hierarchical status of the competitor is known to the test mouse. This is consistent with previous studies indicating that PFC circuits help regulate goal-directed behaviors, partly by storing and retrieving various forms of memory^51–54,65^. For example, in fear conditioning and extinction learning paradigms, the infralimbic PFC is critical for learning and retrieving stimulus-outcome contingencies and overriding previously learned behaviors, acting partly through projections to amygdala-associated inhibitory interneurons^51,52,66–68^. Our findings are consistent with the possibility that an analogous process may be involved in overriding the learned tendency of a subordinate mouse to avoid confrontations with a dominant partner.

Some important limitations should also be noted. First, fiber photometry records the summed activity of all GCaMP-expressing cells at the fiber tip in a single channel. Although we were able to resolve activity time-locked to specific behaviors, suggesting a degree of functional homogeneity in the vmPFC-NAcSh subpopulation, endoscopic recording approaches will be critical for elucidating functional heterogeneity at the single-cell level and would eliminate potential confounds that may arise from recording from fibers of passage in NAcSh in our terminal recording experiments. Second, we used chemogenetic tools to provide sustained inactivation of the vmPFC-NAcSh circuit across the duration of each behavioral test, due in part to the fact that naturalistic social behaviors often occur unpredictably, complicating efforts to deliver temporally specific optogenetic inhibition aligned to a specific behavior. While our targeting for these experiments was primarily in vmPFC, we also cannot rule out the involvement of other areas of mPFC, such as the dorsomedial PFC (dmPFC)^11^, in contributing to our chemogenetic results. Future studies involving optogenetic inhibition of both the vmPFC-NAcSh circuit and other downstream projection targets, possibly in combination with more structured behavioral assessments, will be important for defining temporally precise contributions to behavior. Optogenetic approaches could also be used to further elucidate topological specificity by selectively inhibiting NAcSh terminals, ruling out potential effects of collateral projections on other downstream targets such as the basolateral amygdala^69^. While our chemogenetic inhibition experiments demonstrate that vmPFC-NAcSh activity is necessary for promoting social approach of dominant partners, our chemogenetic and optogenetic gain-of-function experiments suggest that artificial stimulation of this circuit alone is not enough to drive effortful social dominance behavior. These results align with recent studies from Zhou et al.^11^ and Li et al.^70^, who found that stimulation of the vmPFC did not alter social approach behaviors. In contrast, optogenetic activation of dorsomedial PFC neurons increased effortful social dominance behaviors in at least one prior report^11^. Together, these results suggest that coordinated activity in dmPFC neurons and vmPFC-NAcSh projections are critical for regulating approach behaviors in social competitions, but stimulating the vmPFC-NAcSh projection alone is insufficient to drive increased social dominance behavior. Another possibility is that driving social approach behavior may require specific activity patterns within vmPFC-NAcSh projections that have yet to be elucidated, beyond generalized high-frequency activation.

Third, like any social behavior assay, the tube test and open-field social interaction test measure a circumscribed range of social behaviors in a very specific context. Converging results from these two tests define a specific role for the vmPFC-NAcSh circuit in regulating social behavior—namely, initiating social approach and effortful social dominance behaviors when a subordinate mouse encounters a dominant member of an established social hierarchy. However, these findings do not rule out other functional roles. Fourth, we targeted the lateral area of NAcSh, primarily motivated by prior work indicating a role for this region in regulating responses to social stress^43^; however, it will be interesting in future work to explore differential contributions of other areas of NAcSh.

Finally, our results build on prior work to show that the vmPFC-NAcSh circuit not only facilitates but is in fact required for initiating adaptive social approach behavior and supporting stress resilience after repeated bouts of social defeat. Previous work has shown that optogenetic stimulation of NAc-projecting PFC cells increases social interaction behavior after chronic defeat stress^43^. Our chemogenetic inhibition studies show that vmPFC-NAcSh activity is required for initiating social approach behavior following chronic social stress. Interestingly, the effects of chemogenetic inhibition appeared comparable in the stress-susceptible and stress-resilient subgroups, despite the fact that our photometry recordings revealed significantly larger signals during social interaction in stress-resilient individuals compared to stress-susceptible ones. This suggests that vmPFC-NAcSh activity may be engaged in other functions in the open field social interaction test that are not time-locked to social approach but are still important for regulating interaction behavior. Notably, the vmPFC-NAcSh circuit was not engaged during social approach in unstressed control mice, which had no prior experience with dominant CD1 mice, or in stressed individuals approaching a novel (unfamiliar) C57/BL6J mouse. Again, these findings highlight how the vmPFC-NAcSh circuit plays a highly specific, experience-dependent functional role in overriding social avoidance behavior and promoting social approach during social competitions and after repeated social defeats. They parallel our tube test findings in established versus novel competitors, indicating vmPFC-NAcSh activity is sensitive to prior experience establishing the partner’s hierarchical status and regulates social interaction behavior specifically in the context of established hierarchies. Future efforts to define the neurobiological mechanisms that determine individual differences in circuit engagement could provide promising therapeutic avenues for promoting social interaction behavior and stress resilience, both post-hoc and prophylactically^65,71,72^.

## Methods

### Mice

Adults (~postnatal day 60-70) male wildtype C57BL/6 J (Jackson Laboratories) and retired male CD1 breeders aged 4–6 months (Charles River Laboratories) were used for experiments. Mice were housed under climate-controlled conditions on a 12-hour light/dark cycle with ad-libitum access to food and water. All behavioral tasks were run during the light phase. All procedures administered were approved by the Weill Cornell Medicine Institutional Animal Care and Use Committee. All procedures and protocols were in accordance with the 2011 Eight Edition of the National Institutes of Health Guidelines for the Care and Use of Laboratory Animals.

### Stereotaxic injection and optical cannula implantation surgeries

Animals were anesthetized with 1.5% isoflurane and placed into a stereotax (Kopf Instruments). Midline incisions were made down the center of the scalp, and a craniotomy was performed with a dental drill. For fiber photometry vmPFC-NAcSh cell body experiments, AAV-Syn-FLEX-GCaMP6s-WPRE was stereotaxically injected in the ventromedial prefrontal cortex at ML ± 0.35; AP + 1.7; DV −3.2. rAAV2-retro-Cre was injected in the lateral nucleus accumbens shell at ML ± 1.72; AP + 1.25; DV −4.50. A mono fiber-optic cannula (Doric; Quebec, Canada) was placed in the ventromedial prefrontal cortex at ML ± 0.35; AP + 1.7; DV −3.0. For fiber photometry vmPFC-NAcSh terminal experiments, AAV-Syn-GCaMP6s-WPRE.SV40 (Penn Vector P2822) was stereotaxically injected in the ventromedial prefrontal cortex at ML ± 0.35; AP + 1.7; DV −3.2 and a mono fiber-optic cannula (Doric; Quebec, Canada) was placed in the lateral nucleus accumbens shell at ML ± 1.72; AP + 1.25; DV −4.50. Fiber-optic cannulae had a 0.48 refractive index and 400 nm diameter cut to either 3 mm (vmPFC recording) or 4.5 mm (terminal recording) in length using a ruby knife (ThorLabs) and were lowered via a cannula holder (Kopf Instruments) and secured to the skull with dental cement kit (C&B Metabond, Parkell Inc.). For all chemogenetic inhibition experiments, adeno-associated viral vectors (AAV-hsyn-DIO-hM4D(Gi)-mCherry^50^ or AAV-hsyn-DIO-mCherry, 200 nL volume) were injected bilaterally in the vmPFC at ML ± 0.35; AP + 1.7-; DV −3.2 and 200 nL of rAAV2-retro-Cre was injected bilaterally in the NAcSh at ML ± 1.72; AP + 1.25; DV −4.50, yielding selective expression of inhibitory DREADDs or control fluorophore in NAc-projecting vmPFC neurons. pAAV-hSyn-DIO-hM4D(Gi)-mCherry was a gift from Bryan Roth (Addgene viral prep #44362-AAV2; http://n2t.net/addgene:44362; RRID: Addgene_44362). All coordinates were derived from the Mouse Brain Atlas and were relative to Bregma (Paxinos and Franklin, 2004). All injections used a 10 μl Nanofil syringe (WPI, Sarasota, FL) with a 33 gauge blunt metal needle to infuse virus with a microsyringe pump and controller (UMP3, Micro4; Sarasota, FL) at a rate of 50 nL per minute, and kept at the injection site for ~15 min post injection and then slowly withdrawn. Animals’ heads were adjusted to a DV relative difference between Lambda and Bregma ± less than 0.03 mm. Behavioral experiments began after a minimum of 2 weeks to allow for recovery from surgery. All photometry recordings and DREADDs manipulations were conducted at least 3 weeks post injection to allow for maximal viral construct expression. Mice with inaccurate targeting of the viral construct as assessed by post-mortem histological analyses were eliminated from the study.

For all excitation gain-of-function experiments (Supp. Fig 9a–f) animals were bilaterally injected with 200 nl of rAAV2-retro-Cre in the lateral nucleus accumbens shell at ML ± 1.72; AP + 1.25; DV −4.50. For chemogenetic experiments (Supp. Fig 9a–c), animals were bilaterally injected with 200 nl of the excitatory DREADD pAAV-hSyn-DIO-hM4D(Gq)-mCherry into the vmPFC at ML ± 0.35; AP + 1.7-; DV −3.2. For optogenetic experiments seen in Supp. Fig. 9d–f, animals were bilaterally implanted with an optic fiber and bilaterally injected with pAAV-hsyn-DIO-ChR2-mCherry at ML ± 0.35; AP + 1.7-; DV −3.2. For experiments in Supp. Fig 9g–j, animals were injected unilaterally with 200 ul of either AAV-CAG-ChR2-tdTomato or AAV-Ubi-eGFP at 14° angle ML + 0.71, AP + 2.43, DV + 1.67 mm from bregma as described previously^11^. A 400 nm diameter fiber-optic cannula was unilaterally implanted at 400 µm above viral injections.

### Tube test for social dominance behavioral protocol

Tube test training and stable hierarchical determination were adapted from the protocols described in Wang et al., Larrieu et al., and Fan et al.^17,46,65^. Mice were group housed a minimum 4 weeks prior to testing. Mice were first trained to traverse a clear Plexiglas tube (diameter, 3 cm; length, 30 cm) for 3 consecutive days. If mice retreated or were stationary for more than 30 seconds-1 minute, they were gently pushed with the end of a plastic rod. Tubes were cleaned with 70% ethanol solution and dried in between trials in order to clean urine, feces, and eliminate odors. Following training to traverse the tube, social hierarchies per cage each day were evaluated by testing mice in the same cage against each other, round robin style. Wins were classified as occurring when one mouse remains in the tube and the partner mouse fully retreats, with its two rear paws touching the ground outside of the tube. Animals were ranked within cage based upon the number of wins during testing for that day. Round robin testing was performed daily and hierarchies were considered stable when animals ranks remained the same for four days in a row as per Fan et al.^46^. Fiber photometry recordings were obtained only on stable days of testing while chemogenetic manipulations were performed on days 2–4 of testing following an initial day of hierarchy determination (see Supplementary Fig. 2 for data regarding hierarchy changes over time from day 1 to stable). For experiments involving tube test competitions with a novel social partner, subordinate experimental mice were paired with novel (age and weight matched) C57 mice that were ranked dominant within their own cage, and dominant experimental mice were paired with novel (age and weight matched) C57 mice that were ranked subordinate within their own cage. On each day of testing, experimental animals were presented with a new novel partner. Sessions were video-recorded and were scored in an automated manner.

For the experiments in Suppl. Fig. 9a–c, chemogenetic activation was conducted on the day following baseline tube test assessment of social hierarchy where subordinate and dominant groupings were determined. As above, animals received an intraperitoneal injection of CNO or saline at a concentration of 0.5 mg/mL, 1 h before the task. During optogenetic activation experiments, stimulation occurred (10 mW, 20 Hz, 50% duty cycle) in subordinate mice on the day following baseline tube test assessment of social hierarchy in both ChR2 and viral control injected mice. On the following day, dominant mice were stimulated. Optogenentic stimulation began immediately prior to the start of the bout and ended when the bout finished. For the experiments in Supp. Fig. 9g–j, rank 0 mice were stimulated as described in Zhou et al.^11^.

### DeepLabCut automated tube test behavioral scoring

To extract the pose of freely-behaving mice in the tube test assay, we implemented DeepLabCut^49^, an open-source convolutional neural network-based toolbox, to identify the nose and tailbase xy-coordinates of each mouse for each recorded video frame. These coordinates were then used to calculate velocity and position at each time point, as well as classify body push, nose push, resist and retreat epochs. ‘Retreat’ was defined as epochs during which a mouse’s tailbase velocity exceeded a specified negative threshold and the mouse moved a minimum distance in the direction opposite the partner mouse. ‘Body push’ was defined as epochs in which the aggressor nose and tailbase velocities exceeded a specified threshold, and tracked nose positions overlapped for the mouse pair. Additionally, the non-aggressor mouse could not retreat during the push. ‘Nose push’ was categorized in a similar manner, but without the tailbase velocity criterion. ‘Nose push’ behavior was utilized specifically to investigate any differences in push effort between subordinate and dominant animals; ‘Body push’ was the primary type of push behavior used in all other analyses and is referred to simply as ‘push’ behavior throughout the manuscript.

### Chemogenetic manipulation of tube test behavior

For the experiments in Fig. 2, chemogenetic manipulations were conducted on the day following a baseline tube test assessment of social hierarchy where subordinate and dominant groupings were determined. Animals received Clozapine-N-Oxide (CNO) (Enzo Life Sciences) dissolved in 0.9% saline at a concentration of 0.5 mg/mL and injected intraperitoneally at 0.01 mL/g body weight for a final dose of 5 mg/kg, 1 h before the task. Partners, dominant or subordinate depending on the experiment received saline injections. In addition, to control for off-target effects of CNO, a separate cohort of mice received viral control injections at the time of surgery and then received CNO or saline on testing day.

### Territory urine marking assay behavioral protocol

We used the Urine Marking Assay to validate social hierarchies identified in the tube test (Supplementary Fig. 2e, f). As per standard protocols^10,48^, dominant and subordinate pairs (as determined by the tube test described above) from within the same home cage were placed into a modified chamber (10” × 10” × 10”) with open grid flooring and a wire mesh divider down the center allowing animals to see and smell each other. Filter paper was placed under the open flooring. Animals were in sensory contact with each other in this chamber for 2 h. At the termination of the 2 h, filter papers were viewed with a UV light source to analyze the spatial distribution of urine deposits from the animal pairs. Trained investigators who were blinded to the hierarchical status or experimental condition of the test mice then scored the number of deposits, the area occupied by each deposit, and the location of the urine deposits with respect to the partner mouse (i.e. adjacent to the barrier separating the mice or away from the barrier). For each pair of mice, an individual was classified as dominant if it produced a larger number of deposits over a greater territory (area), including areas adjacent to the barrier. A small number of pairings (<10%) with no determinable dominant partner were excluded from the contingency table in Supp. Fig. 2f. Fisher’s exact test was used to test for statistical significance and evaluate whether hierarchical classifications in the tube test predicted hierarchical classifications in urine marking assay.

### Chronic social defeat stress (CSDS) behavioral protocol

Chronic social defeat stress was carried out as described in Golden et al.^55^. To summarize, in each social defeat session, the test mouse was introduced into the home cage of an aggressive CD1 mouse where they were physically defeated for a 5–10 min period. After the physical defeat session, experimental animals were kept in sensory contact with aggressors for a 24 h period. This process was repeated for 10 days with a new aggressor each day. After the last defeat session and sensory contact period, stressed animals were solo housed for a 24-hour period. After which, they were tested in the open field social interaction task. A two trial behavioral task where in trial one, experimental mice were introduced to an empty box with an empty perforated chamber (10 cm × 6.5 cm × 42 cm) at the center of the far end of the box and were allowed to explore for 2.5 min. Experimental mice are then removed for 30 s during which the empty chamber is swapped for a perforated chamber containing a novel, non-aggressive CD1 social partner. The experimental mice were then re-introduced to the box for trial two and are allowed to explore for another 2.5 min. During both trials, exploratory behavior was tracked by Ethovision 11.5 XT (Noldus, Information Technology), specifically identifying exploration into the social interaction zone surrounding the perforated chamber (a 14 cm × 24 cm area centered around the chamber). Modifications to this standard task included adding a third trial to the open field social interaction task, in which stressed and control mice were presented with an age-matched novel (unfamiliar) C57/BL6 partner mouse to test whether social avoidance and circuit activity effects were specific to the CD1 mouse strain used in the social defeat bouts. Fiber photometry experiments were conducted during this social interaction task. As in previous studies^59^, stressed animals were divided into susceptible and resilient subgroups based on a social avoidance score, which was calculated by averaging the Z-scored social interaction zone ratio (social zone time in trial two vs. trial one), Z-scored corner zone ratio (corner zone time in trial two vs trial one), and Z-scored interaction zone duration and corner zone duration during trial two of the open field social interaction task. As described in Anacker et al.^59^, the social interaction ratio Z-score and social interaction duration Z-score were multiplied by −1 before averaging all Z-scores, such that for all four measures, a highly positive score would indicate social avoidance behavior. Individuals with scores above zero were classified as stress susceptible (exhibiting social avoidance), while individuals with scores below zero were classified as stress resilient (not exhibiting social avoidance).

Chemogenetic manipulation experiments were conducted 24 h after the baseline open-field social interaction task. For these experiments, stressed and unstressed animals (for both the hM4Di and viral control groups) received injections of CNO (Enzo Life Sciences, dose and preparation as described above) one hour prior to OFSI behavioral testing.

### Forced interaction task behavioral protocol

The Forced Interaction Task (FIT) was conducted in two trials where the experimental animal was confined to a small center chamber (10 cm × 6.5 cm × 42 cm) and a non-aggressive CD1 interaction partner, chosen in the same manner as for the open field interaction task^55^, was in the surrounding open field. During trial one which was 2.5 min long, there was no interaction partner in the periphery in order to record baseline photometry signal. Trial two was also 2.5 min long, and the CD1 interaction partner was introduced into the periphery. The task was video-recorded in Ethovision 11.5 XT (Noldus, Information Technology) and CD1 approaches and withdrawals from the interaction chamber (10 cm × 6.5 cm × 42 cm) were hand-scored using behavioral recording software CowLog (http://cowlog.org/). Scorers were blinded to mouse experimental group.

### Sucrose preference and elevated plus maze tasks

For a subset of mice, sucrose preference and elevated plus maze tasks were carried out twice; (1) 24 h before chronic social defeat stress and (2) within 24 h after the social interaction task. During the elevated plus maze task, mice were given five minutes to explore the open and closed arms. The total time spent in the open arms was recorded. During the sucrose preference task, mice were given 6 hours to drink either water or one percent sucrose in water. Mice were given access to food throughout the task. Changes in bottle weight were recorded and sucrose preference was calculated. Schematic diagrams were generated via BioRender^73^.

### Fiber photometry: data acquisition

Fiber photometry^44,74^ was performed to measure calcium-dependent activity dynamics during tube test and social interaction behavior. To excite GCaMP6s, light from a 470 nm LED (Thorlabs, M470F3) modulated at a frequency of 521 Hz was passed through a filter (Semrock, FF02-472/30), reflected by a dichroic (Semrock, FF495-Di03) and coupled to a 0.48 NA, 400 μm core optic fiber patch cord (Doric). Emitted fluorescence traveled back through the patch cord, passed through the dichroic, a filter (Semrock, FF01-535/50), and was focused onto a photodetector (Newport, Model 2151). The modulated signal passed from the photodetector to a RP2.1 real-time processor (Tucker Davis Technologies) where it was demodulated and low-pass filtered using a corner frequency of 15 Hz. For cell body recordings, an isosbestic channel was utilized; this consisted of an additional excitation of GCaMP6s using a 405 nm LED (Thorlabs, M405F1) modulated at a frequency of 211 Hz, which passed through a filter (Thorlabs, FB405-10) and was reflected by a dichroic (Thorlabs, DMLP425R) that allowed the 470 nm channel to pass through. Both of these stimulation sources were reflected by an additional dichroic (Semrock, FF495-Di03) and coupled into the patch cord as described above. TTL pulses denoting the start of behavioral trials were passed to the processor in real-time for alignment of calcium signals to behavioral measures.

### Fiber photometry: data analysis

Data were analyzed using custom MATLAB (Mathworks) scripts. For cell body recordings, fluorescence signals were normalized (transformed to ΔF/F) using an isosbestic control channel by least-squares fitting the control channel to the data and then subtracting the fit control signal from the data and dividing by this control data ((data—fit control)/(fit control). For terminal recordings, signals were normalized by taking the median value of the signal during a 10 s (± 5 s) sliding window around each data point, subtracting this median from the data point and then dividing by the median. Following normalization, animals in which the difference in ΔF/F between the 95th percentile and 5th percentile of signal during recording was less than 1% ΔF/F were excluded from analysis due to a lack of fluorescent signal variation, usually indicating poor GCaMP6 expression or inaccurate fiber placement.

For the tube test data, to ensure analysis was not confounded by signal changes related to handling the mouse at it was placed in the tube, only recordings from tube test bouts lasting longer than 15 s were included in further analysis. Recordings were time-locked to behavioral events of interest (e.g., push, resist, or retreat) and a baseline signal was calculated as the average fluorescence value from the second prior to the behavior of interest. Signal values were then shifted by this baseline value to detect changes in signal due to the behavioral event. Average time-locked traces represent the mean signal from −1.0 s to 5.0 s following the behavior of interest. To test for differences between experimental groups in circuit activity associated with a particular behavior, we quantified the mean amplitude of the signal during the 4 s following the behavior of interest in each group.

For analysis of social behavior tests following CSDS (OFSI, FIT), recordings were time locked to behavioral events of interest. Behavioral events preceded by a prior event in the last 3 s were excluded from analysis to prevent confounds related to previous signal changes due to the relatively slow dynamics of the calcium sensor. A baseline signal was calculated as the average fluorescence value from −1.5 s to −1.0 s prior to the behavior of interest. Signal values were then shifted by this baseline value to detect changes in signal due to the behavioral event. Average time-locked traces represent the mean signal from −1.5 s prior to 5.0 s following the behavior of interest. Between-group differences in activity associated with a particular behavior were quantified in terms of the mean amplitude of the signal from the onset of the behavior (0 s) to 4.0 s following the behavior. To test whether circuit activity changed significantly upon initiation of a given behavior, we calculated the average signal during a period spanning −1.0 to 0.0 s prior to the behavior (pre) and compared it with the average signal during a period spanning 0.0 s to 1.0 s following the behavior (post).

### Statistical analyses

Statistical analyses were conducted in Prism 7.0d (GraphPad software) or MATLAB (Mathworks) using custom scripts. As described in the figure legends, we used Welch’s *t* tests (insensitive to inequality of variance) and one- or two-way ANOVAs as appropriate, with Bonferroni corrections for multiple comparisons in post-hoc tests. In addition, for our fiber photometry data analyses, we primarily used linear mixed-effects models in which mouse identity was modeled as a random effect, to account for the fact that in each experiment, repeated fiber photometry measures were obtained from each mouse. Data were checked and confirmed to meet the assumptions of the statistical test being used. All reported *p* values are two-tailed, and all data are presented as the mean ± s.e.m. All outliers were determined using a Grubb’s test, with Alpha = 0.05, and excluded from subsequent analyses. Sample sizes were guided by previously published literature.

### Fixation, sectioning and histology

Fixation and sectioning were conducted in order to confirm injection targets and expression. Following the termination of behavioral testing, mice were anesthetized with an injection of euthasol (150 mg/kg, I.P.) and brains were fixed with a 4% paraformaldehyde via aortic arch perfusion followed by cryoprotection in 30% sucrose in 0.1 M 251 phosphate buffer (PB) at 4 °C for 72 h. Coronal sections were cut on a cryostat (20 μm). For cell body photometry tissue, GCaMP6s expression cells were identified via immunohistochemistry targeting GFP (primary—anti-GFP Abcam (1:200, ab13970); secondary—goat anti-chicken Alexa 488 (1:1000, Thermofisher, A-11039)). For DREADD experiments, hM4Di-expressing cells were identified via immunohistochemistry targeting mCherry (1:200, anti-mCherry Abcam (ab167453); secondary—goat anti-rabbit Alexa 488 (1:1000, Abcam, ab150077)). Sections were mounted using VECTASHIELD Antifade Mounting Medium with DAPI for fluorescent microscopy (Vector). Sections were imaged using an epifluorescent microscope 256 (Leica DM550B with Leica Application Suite Advanced Fluorescence 3.0.0 build 8134 257 software, Leica Microsystems).

### Reporting summary

Further information on research design is available in the Nature Portfolio Reporting Summary linked to this article.

## Supplementary information

Supplementary Information

Description of Additional Supplementary Files

Supplementary Movie 1

Supplementary Movie 2

Supplementary Movie 3

Reporting Summary

## Data Availability

The main data supporting the results in this study are provided with this paper (please see attached source data). Pre-processed data are also available from the corresponding authors upon reasonable request. Source data are provided with this paper.

## Source data

Source Data

## Code availability

Fiber photometry data collection and analyses were based upon previously published work^44^. Automated behavioral analyses utilized the DeepLabCut^49^ toolbox.
