# Supplementary material for: Regulation of social interaction in mice by a frontostriatal circuit modulated by established hierarchical relationships
Source: Nat Commun. 2023 Apr 29;14:2487. doi: 10.1038/s41467-023-37460-6 (PMC10148889; doi:10.1038/s41467-023-37460-6)

Regulation of social interaction in mice by a frontostriatal circuit modulated by established hierarchical relationships

Robert N. Fetcho^, Baila S. Hall^, David J. Estrin^, Alexander P. Walsh, Peter J. Schuette, Jesse Kaminsky, Ashna Singh, Jacob Roshgodal, Charlotte C. Bavley, Viraj Nadkarni, Susan Antigua, Thu N. Huynh, Logan Grosenick, Camille Carthy, Lauren Komer, Avishek Adhikari, Francis S. Lee, Anjali M. Rajadhyaksha*, Conor Liston*

^These authors contributed equally to this work.

*Correspondence to col2004@med.cornell.edu or amr2011@med.cornell.edu.

Supplementary Material

Supplementary Figure 1. Representative optical fiber placement for vmPFC-NAcSh cell body photometry experiments. Schematic showing representative fiber tip locations for recordings from cell bodies in vmPFC projecting to NAcSh (N = 13). Images modified from the Allen Reference Atlas – Mouse Brain.

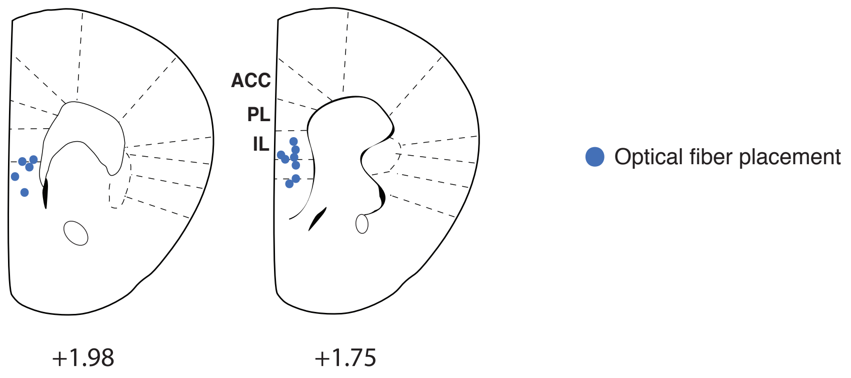

Supplementary Figure 2. Determination of cage hierarchies using the tube test behavior.

- **a.** Timeline of tube test behavioral testing to determine stable within-cage hierarchies.
- **b.** Example cage rankings from three cages across repeated daily tube test behavior.
- **c.** Animals' hierarchical ranks determined on Day 1 of the tube test were significantly correlated with their final stable rank following repeated testing. N = 34 mice, 9 cages; Spearman $\rho = 0.6874$; $^{****}p < 0.0001$.
- **d.** Animals' classification as either subordinate (rank 3 or 4) or dominant (rank 1 or 2) on day 1 of tube test was significantly associated with their final classification when stable ranks were achieved. N = 34 mice, 9 cages; Fisher's exact test $^{**}p = 0.005$.
- **e.** Urine marking assay schematic and sample images of urine patterns from dominant and subordinate pairs.
- **f.** An animal's classification as either subordinate or dominant in the tube test was significantly associated with their classification via the urine marking assay. N = 38 animals, 2 experiments; Fisher's exact test $^{***}p < 0.001$.

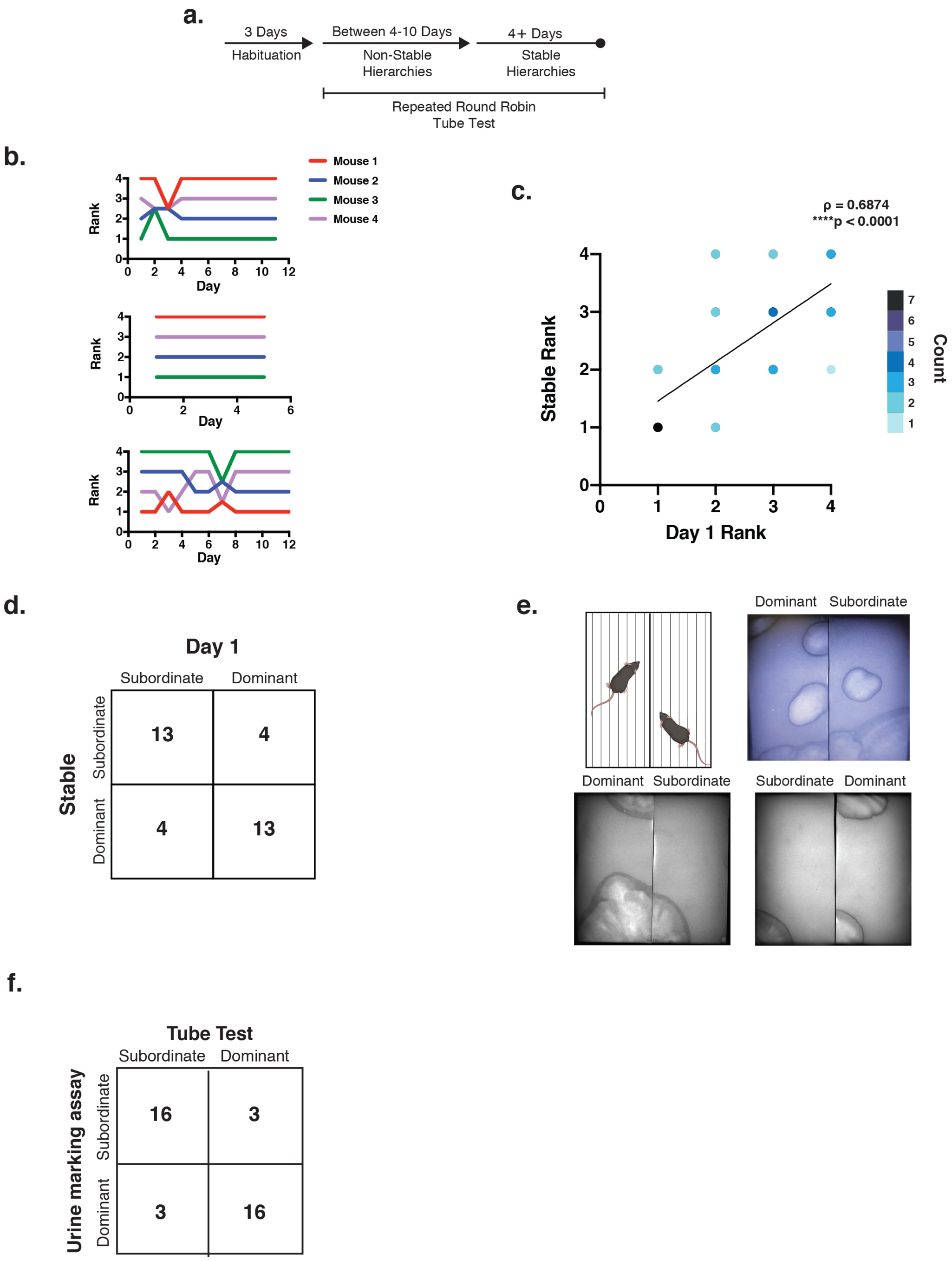

Supplementary Figure 3. DeepLabCut automated scoring of tube test behaviors.

- **a.** Example of DeepLabCut simultaneous tracking of nose and tail base of both animals during a tube test bout. Push, resist, and retreat behaviors were automatically identified based on characteristic changes in the animals' position in the tube as described in the Online Methods.
- **b.** Push rates of tube test behavioral bouts scored by DeepLabCut were significantly correlated with hand-scored push rates of the same bouts by experienced scorers. N = 24 bouts; Spearman ρ = 0.6125; **p = 0.002.
- **c.** Resist rates of tube test behavioral bouts scored by DeepLabCut were significantly correlated with hand-scored retreat rates of the same bouts by experienced scorers.
  N = 24 bouts; Spearman ρ = 0.5942; **p = 0.002
- **d.** Retreat rates of tube test behavioral bouts scored by DeepLabCut were significantly correlated with hand-scored resist rates of the same bouts by experienced scorers. N = 24 bouts; Spearman ρ = 0.6989; ***p = 0.0001.

a.

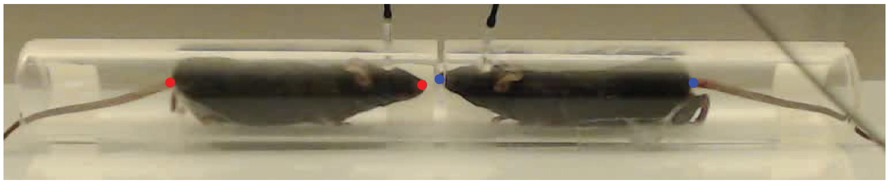

b.

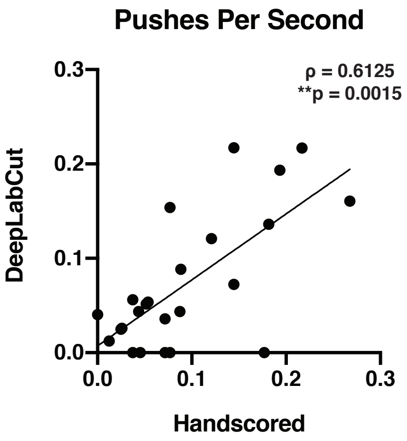

c.

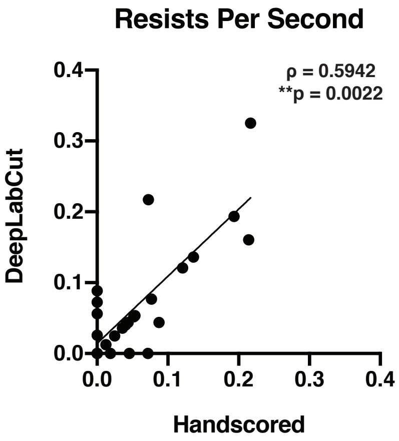

d.

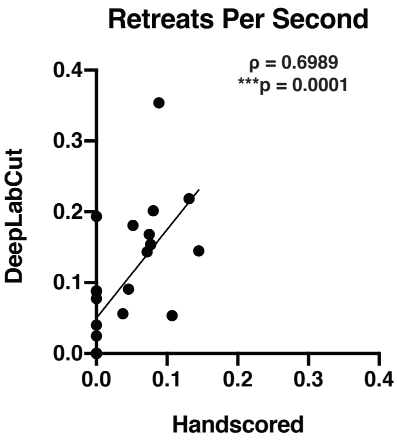

Supplementary Figure 4. vmPFC-NAcSh activity levels during push behavior as a function of hierarchical rank differences.

- **a.** Average vmPFC-NAcSh activity during push behavior grouped by the difference in rank between the recorded experimental mouse and their partner. A rank difference of 3 indicates the recorded animal is the most subordinate in the cage and is pushing against the most dominant in the cage. A rank differences of –3 indicates the recorded animal is the most dominant in the cage and is pushing against the most subordinate in the cage. Linear mixed effects model with rank difference as a fixed effect and subject as a random effect $T(93) = 1.81$, $p = 0.07$ for main effect of rank difference on mean amplitude ($N = 95$ bouts from 16 animals). Error bars presented as mean +/- SEM.
- **b.** In order to test if the same experimental animals show differential vmPFC-NAcSh activity when facing either more dominant or subordinate partners, we visualized activity during push behavior for rank 2 (b) and rank 3 (c) individuals. Mean (+/– SEM) photometry trace of vmPFC-NAcSh circuit activity time-locked to the initiation of pushes for rank 2 mice encountering a dominant competitor (blue, rank 1 animals) and rank 2 mice encountering a subordinate competitor (red, rank 3 or 4 animals).
- **c.** Mean (+/– SEM) photometry trace of vmPFC-NAcSh circuit activity time-locked to the initiation of pushes for rank 3 mice encountering a dominant competitor (blue, rank 1 or 2 animals) and rank 3 mice encountering a subordinate competitor (red, rank 4 animals). The traces in panels b and c indicate that vmPFC-NAcSh activity varies within the same individuals, dependent upon the hierarchical status of the partner.

a.

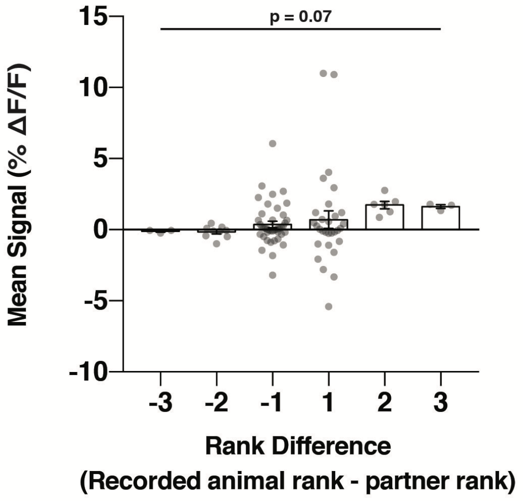

b.

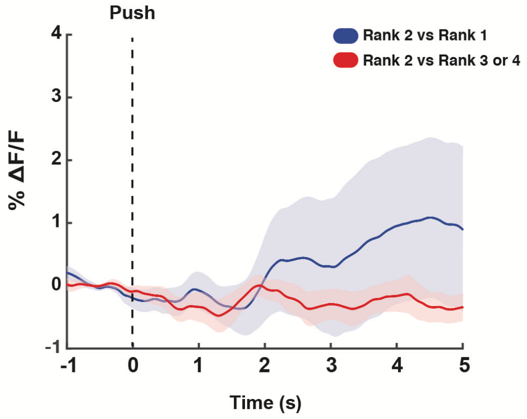

c.

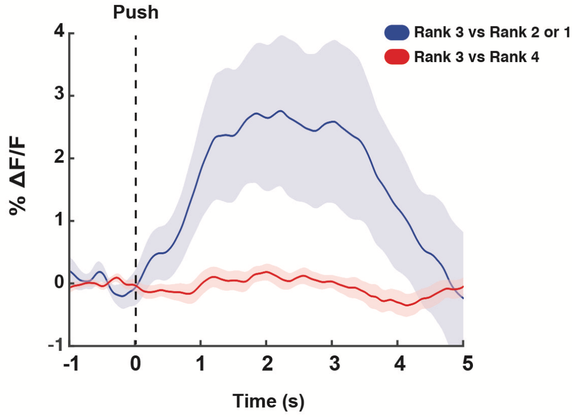

Supplementary Figure 5. Retreat and resist vmPFC-NAcSh activity heatmaps.

- **a.** Heat map showing vmPFC-NAcSh circuit activity during retreat events for all subordinate and dominant animals. Each row is an individual retreat event and rows are organized by animal (note one dominant animal did not have any retreat events).
- **b.** Heat map showing vmPFC-NAcSh circuit activity during resist events for all subordinate and dominant animals. Each row is an individual resist event and rows are organized by animal.

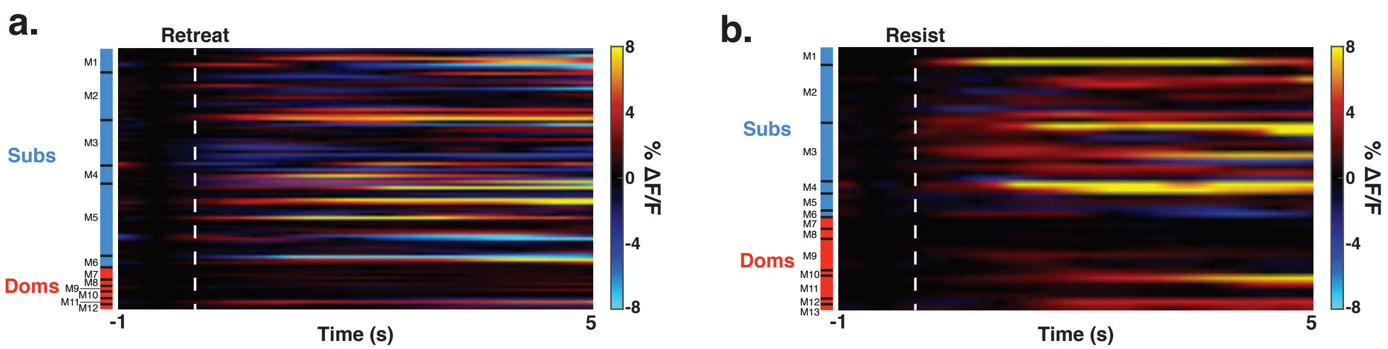

Supplementary Figure 6. vmPFC-NAcSh activity differences during push behavior are not related to differences in effort-level of push behavior or locomotion.

a. The duration of pushes did not differ between subordinates pushing against dominant partners compared to dominants pushing again subordinate partners, indicating that differences in push-related activity were not attributable to differences in the duration of pushes initiated by subordinate vs. dominant mice. Welch's Unpaired t-test, $T_{(85.46)}=0.67$, $p = 0.50$. (N = 39 Sub v Dom; 73 Dom v Sub). Error bars presented as mean +/- SEM.

b. Mean vmPFC-NAcSh photometry signal during push behavior plotted in relation to the duration of the push reveals no significant correlation, 16 mice, 4 cages, N = 110 events, Pearson's correlation r = 0.11, p = 0.27.

c. In addition to "body pushes" (the push behavior our primary analysis was based upon) we also identified "nose pushes" as an alternative, lower-effort type of push behavior (see Online Methods). We found that the fraction of "body pushes" performed compared to all pushing behavior (body + nose pushes) did not differ between subordinates pushing against dominant partners compared to dominants pushing again subordinate partners, indicating that differences in push-related activity were not attributable to differences in the likelihood of engaging in high-effort "body pushes" vs. lower-effort "nose pushes". Welch's Unpaired t-test, $T_{(93.14)}=1.02$, $p = 0.31$. (N = 46 Sub v Dom; 62 Dom v Sub). Error bars presented as mean +/- SEM.

d. vmPFC-NAcSh photometry signal plotted in relation to the velocity of the recorded animal during the tube test reveals no correlation between velocity and circuit activity during tube test bouts. N = 16 mice, 4 cages, 4182 bins; Pearson's correlation p = 0.182.

e. vmPFC-NAcSh photometry signal plotted in relation to the velocity of the recorded animal during movement in an open field reveals no correlation between velocity and circuit activity. N = 42 mice, 6671 bins; Pearson's correlation p = 0.294. Together, the results in panels **d** and **e** indicate that vmPFC-NAcSh activity changes were not related to locomotion but rather were specific to the initiation of effortful push behaviors in subordinate mice encountering a dominant partner.

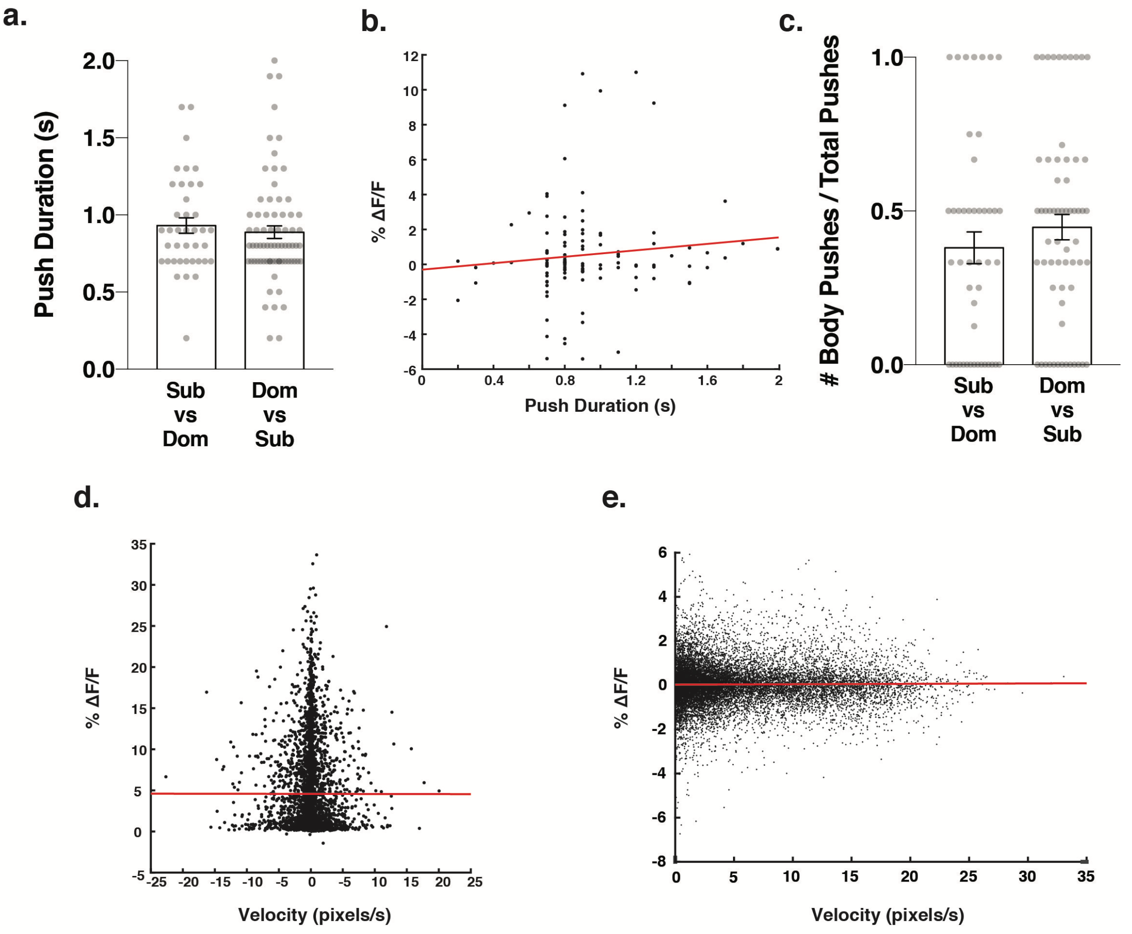

Supplementary Figure 7. vmPFC-NAcSh terminal recordings during tube test replicate vmPFC-NAcSh cell body recording findings.

- **a.** Experimental schematic, including surgical strategy for viral injection and fiber optic cannula implantation for recording activity from ventromedial prefrontal cortex (vmPFC) axon terminals projecting to the nucleus accumbens shell (NAcSh). Created with BioRender.com
- **b.** GCaMP6s expression in vmPFC cell bodies (4x) and in NAcSh terminals (4x, 20x inset) in a representative animal (scale bar=100 μm). Representative GCaMP6s expression range of focal injections in the vmPFC at different bregma locations. Representative optic fiber placements in the NAcSh (N = 17). Images modified from the Allen Reference Atlas – Mouse Brain.
- **c.** Mean (+/– SEM) photometry trace of vmPFC-NAcSh circuit activity time-locked to the initiation of pushes for subordinate mice encountering a dominant competitor (blue) and dominant mice encountering a subordinate competitor (red).
- **d.** Trending increase in mean amplitude of vmPFC-NAcSh terminal activity (mean signal averaged over a window spanning 2s following push initiation) in subordinate mice encountering a dominant competitor, compared to dominant mice encountering a subordinate competitor. Linear mixed effects model, $T(36) = -1.993$, $p=0.053$ for main effect of experimental condition on mean amplitude (N = subordinate group: 26 push events from 6 mice; dominant group: 12 push events from 5 mice). Error bars presented as mean +/- SEM.
- **e.** Mean (+/– SEM) photometry trace time-locked to the initiation of retreat behaviors.
- **f.** No group difference in vmPFC-NAcSh terminal activity associated with retreat behaviors. Linear mixed effects model, $T(20) = -0.39$, $p=0.698$ (N = subordinate group: 17 retreat events from 5 mice; dominant group: 5 retreat events from 2 mice). Error bars presented as mean +/- SEM.
- **g.** Mean (+/– SEM) photometry trace time-locked to the initiation of resist behaviors.
- **h.** No group difference in vmPFC-NAcSh terminal activity associated with resist behaviors. Linear mixed effects model, $T(66) = 0.49$, $p=0.624$ for main effect of experimental condition on mean amplitude (N = subordinate group: 29 resist events from 7 mice; dominant group: 39 resist events from 6 mice). Error bars presented as mean +/- SEM.

Supplementary Figure 7 on next page

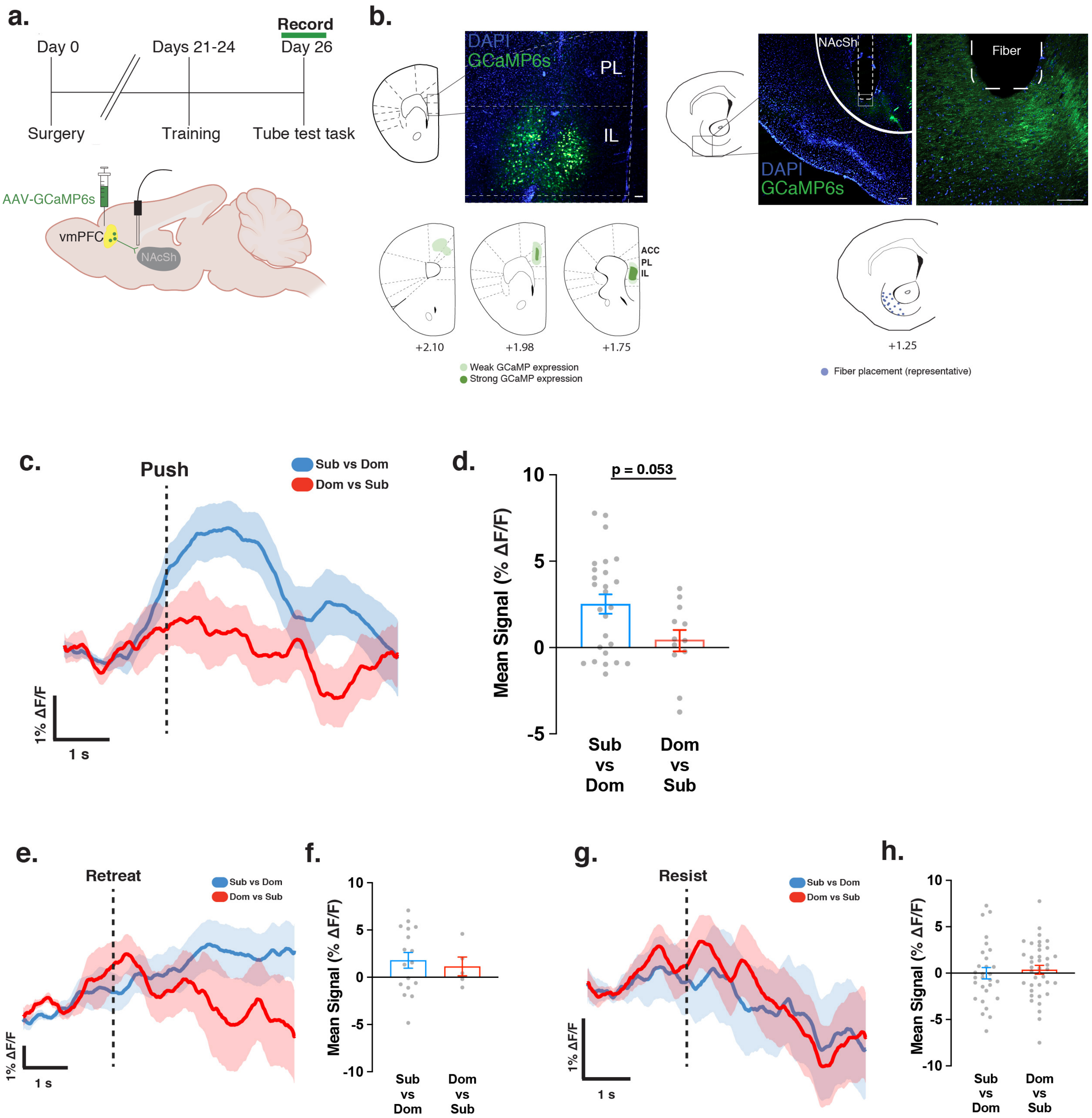

a.
Record
Day 0
Days 21-24
Day 26
Surgery
Training
Tube test task
AAV-GCaMP6s
vmPFC
NAcSh
b.
DAPI
GCaMP6s
PL
IL
NAcSh
Fiber
ACC
+2.10
+1.98
+1.75
+1.25
Weak GCaMP expression
Strong GCaMP expression
Fiber placement (representative)
c.
Push
Sub vs Dom
Dom vs Sub
1% ΔF/F
1 s
d.
p = 0.053
Mean Signal (% ΔF/F)
Sub vs Dom
Dom vs Sub
e.
Retreat
f.
g.
Resist
h.

Supplementary Figure 8. Behavioral effect of inhibiting vmPFC-NAcSh activity was specific to push rates and did not alter other behaviors in subordinate or dominant animals. For **b.— k.**, two-way repeated measures ANOVA were performed with Holm-Bonferroni two-sided t-tests. Error bars indicate mean +/- SEM.

a. Schematic indicating the percentage of animals in chemogenetic experiments (Fig. 2 and Fig. 4) that expressed hM4Di within specified shaded areas. Images modified from the Allen Reference Atlas – Mouse Brain.

b. – f. For subordinate mice expressing hM4Di or viral control versus dominant mice, the **b.** percentage of wins ($F(1,20) = 0.061$, $p>0.05$, N = 8 hM4Di animals, 14 Control animals; 2 experiments), **c.** duration of bouts ($F(1,94) = 0.22$, $p>0.05$, N = 40 hM4Di bouts, 55 Control bouts; 2 experiments), **d.** rate of retreats ($F(1,94)=6.273$, $p>0.05$, N = 40 hM4Di bouts, 56 Control bouts; 2 experiments), **e.** rate of resists ($F(1,94) = 1.219$, $p>0.05$, N= 40 hM4Di bouts, 56 Control bouts; 2 experiments) and **f.** fraction of pushes resisted ($F(1,79)=1.02$, $p>0.05$, N = 22, 29 hM4Di bouts, 13, 19 Control bouts; 2 experiments[#]) were not significantly altered with CNO injection compared to mice injected with saline.

g. – i. For dominant mice expressing hM4Di or viral control versus subordinate mice, the **g.** percentage of wins ($F(1,20) = 0.016$, $p>0.05$, N = 10 hM4Di animals, 12 Control animals; 2 experiments), **h.** duration of bouts ($F(1,78) = 0.005$, $p>0.05$, N= 40 hM4Di bouts, 39 Control bouts; 2 experiments), and **i.** rate of retreats ($F(1,78)=0.11$, $p>0.05$, N = 40 hM4Di bouts, 40 Control bouts; 2 experiments) were not significantly altered with CNO injection compared to mice injected with saline.

j. Resist rates for dominant animals expressing hM4Di or viral control versus subordinate animals were higher among mice injected with CNO ($F(1,78) = 19.5$, ****$p<0.001$, N = 40 hM4Di bouts, 40 control bouts; 2 experiments, hM4Di CNO vs hM4Di saline: $t(78)=4.516$, ****$p<0.001$).

k. The fraction of pushes by a subordinate partner resisted by dominant animals expressing hM4Di or viral control versus subordinate animals showed no significant difference after CNO treatment ($F(1,65)=4.293$, $p>0.05$, N = 15, 18 hM4Di bouts, 25, 11 Control bouts; 2 experiments[#]).

l. Saline versus CNO injection did not affect distance traveled, Welch's Unpaired t-test, $T_{(42.43)}=0.49$, $p = 0.624$. (N = 22 animals saline; 22 animals CNO).

[#]only bouts in which the partner performed a push behavior were included in these analysis

Supplementary Figure 8 on next page

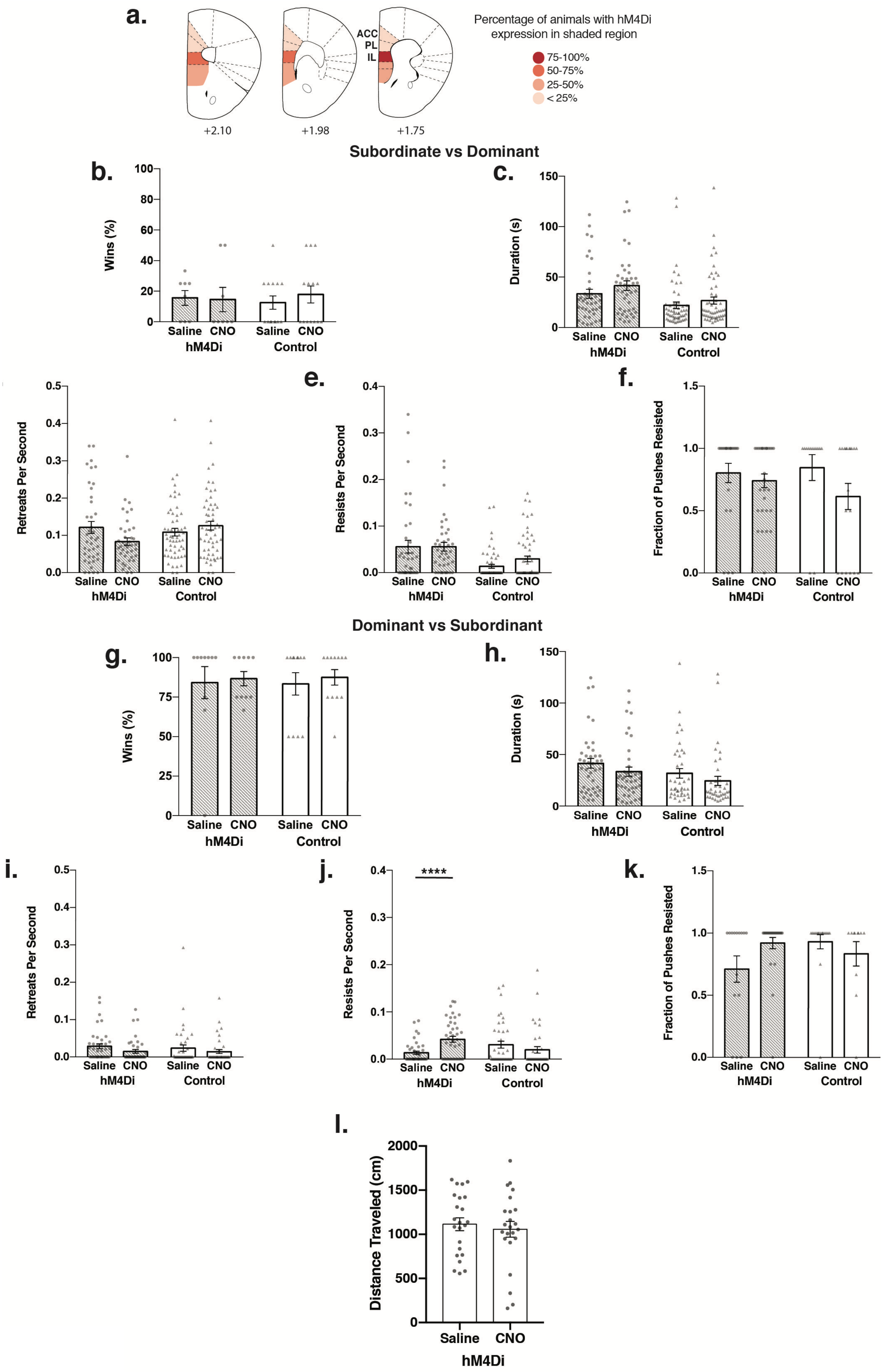
a.
Percentage of animals with hM4Di expression in shaded region
75-100%
50-75%
25-50%
< 25%
ACC
PL
IL
+2.10
+1.98
+1.75
Subordinate vs Dominant
b.
Wins (%)
c.
Duration (s)
e.
Resists Per Second
f.
Fraction of Pushes Resisted
Retreats Per Second
Saline CNO Saline CNO
hM4Di Control
Dominant vs Subordinant
g.
Wins (%)
h.
Duration (s)
i.
Retreats Per Second
j.
Resists Per Second
****
k.
Fraction of Pushes Resisted
l.
Distance Traveled (cm)
Saline CNO
hM4Di

Supplementary Figure 9. Activation of vmPFC-NAcSh during tube test did not alter push behavior.

- **a.** Timeline of vmPFC-NAcSh chemogenetic activation experiment during tube test.
- **b.** CNO did not alter push rate compared with saline in subordinate mice expressing hM3Dq in vmPFC-NAcSh cell bodies facing dominant partners. Paired t-test t(37) = 0.081, p = 0.94 (N=38 bouts in 11 subordinate test mice). Error bars presented as mean +/- SEM.
- **c.** CNO did not alter push rate compared with saline in dominant mice expressing hM3Dq in vmPFC-NAcSh cell bodies facing subordinate partners. Paired t-test t(37) = 1.495, p = 0.14 (N=38 bouts in 8 dominant test mice). Error bars presented as mean +/- SEM.
- **d.** Timeline of vmPFC-NAcSh optogenetic stimulation experiment during tube test behavior.
- **e.** Optogenetic stimulation did not alter push rate during tube test in subordinate mice facing dominant partners. Repeated measures two way ANOVA (N=21 hM4Di bouts from 6 mice, N=26 control bouts from 7 mice), no interaction F(1,45)=0.009, p=0.92. Error bars presented as mean +/- SEM.
- **f.** Optogenetic stimulation did not alter push rate during tube test in dominant mice facing subordinate partners. Repeated measures two way ANOVA (N=25 hM4Di bouts from 7 mice, N=26 Control bouts from 7 mice), no interaction F(1,49)=0.74, p=0.39. Error bars presented as mean +/- SEM.
- **g.** Replicating Zhou et al., 2017,[11] animals were injected unilaterally with either *AAV-CamKII-ChR2-mCherry* or *AAV-Ubi-eGFP* into the dmPFC in order to optogenetically activate dmPFC neurons during tube test competitions as in prior work. The replication consisted of one mouse cohort ran once. See Methods for details.
- **h.** *Top*: Example of mice interacting during the tube test. *Bottom*: Example rank dynamics of ChR2 cage. For this cage, the rank 0 mouse was stimulated on session 12.
- **i.** Average rank change for Rank 0 mice (Two-sided unpaired t-test t(13)=1.38, p=0.09).
- **j.** Average difference in push rate for stimulation trials in ChR2-injected vs. GFP-injected mice (Two-sided unpaired t-test t(25)=2.45, **p=0.009). Error bars indicate average +/- sem.

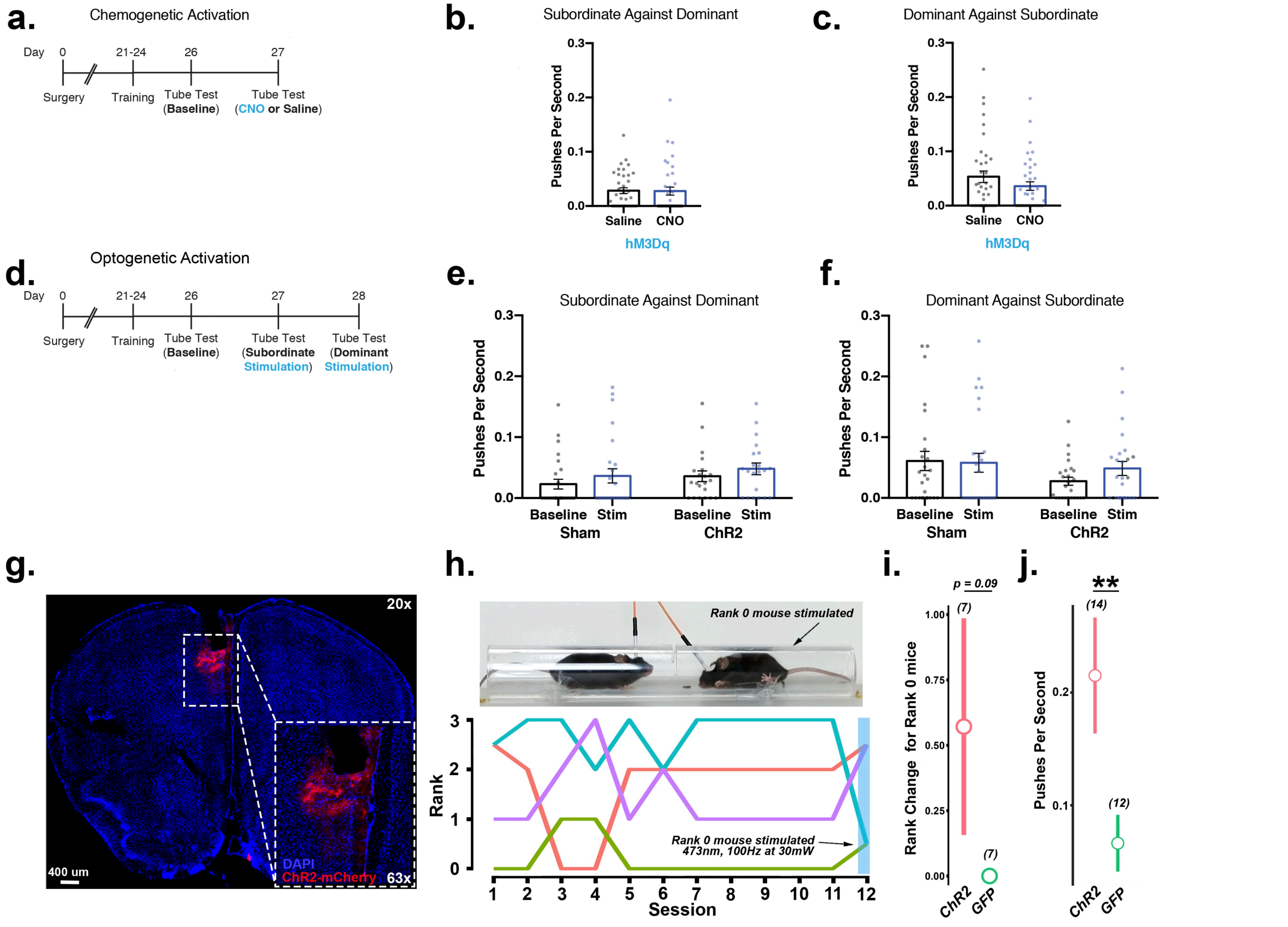

Supplementary Figure 10. vmPFC-NAcSh activity during retreat and resist behaviors in tube test with novel partner.

- **a.** Mean (+/– SEM) photometry trace of vmPFC-NAcSh circuit activity time-locked to the initiation of retreats for subordinate mice encountering a novel dominant competitor (blue) and dominant mice encountering a novel subordinate competitor (red).
- **b.** No group difference in vmPFC-NAcSh circuit activity associated with retreat behavior against novel partners. Linear mixed effects model, $T(105) = -0.47$, $p=0.64$ for main effect of experimental condition on mean amplitude (N = subordinate group: 68 retreat events from 8 mice; dominant group: 39 retreat events from 8 mice).
- **c.** Mean (+/– SEM) photometry trace of vmPFC-NAcSh circuit activity time-locked to the initiation of resists for subordinate mice encountering a novel dominant competitor (blue) and dominant mice encountering a novel subordinate competitor (red).
- **d.** No group difference in vmPFC-NAcSh circuit activity associated with resist behavior against novel partners. Linear mixed effects model, $T(40) = -0.17$, $p=0.866$ for main effect of experimental condition on mean amplitude (N = subordinate group: 29 resist events from 7 mice; dominant group: 13 resist events from 5 mice – note: 1 subordinate and 3 dominant mice in cohort did not have any resist events during their bouts with novel partners).

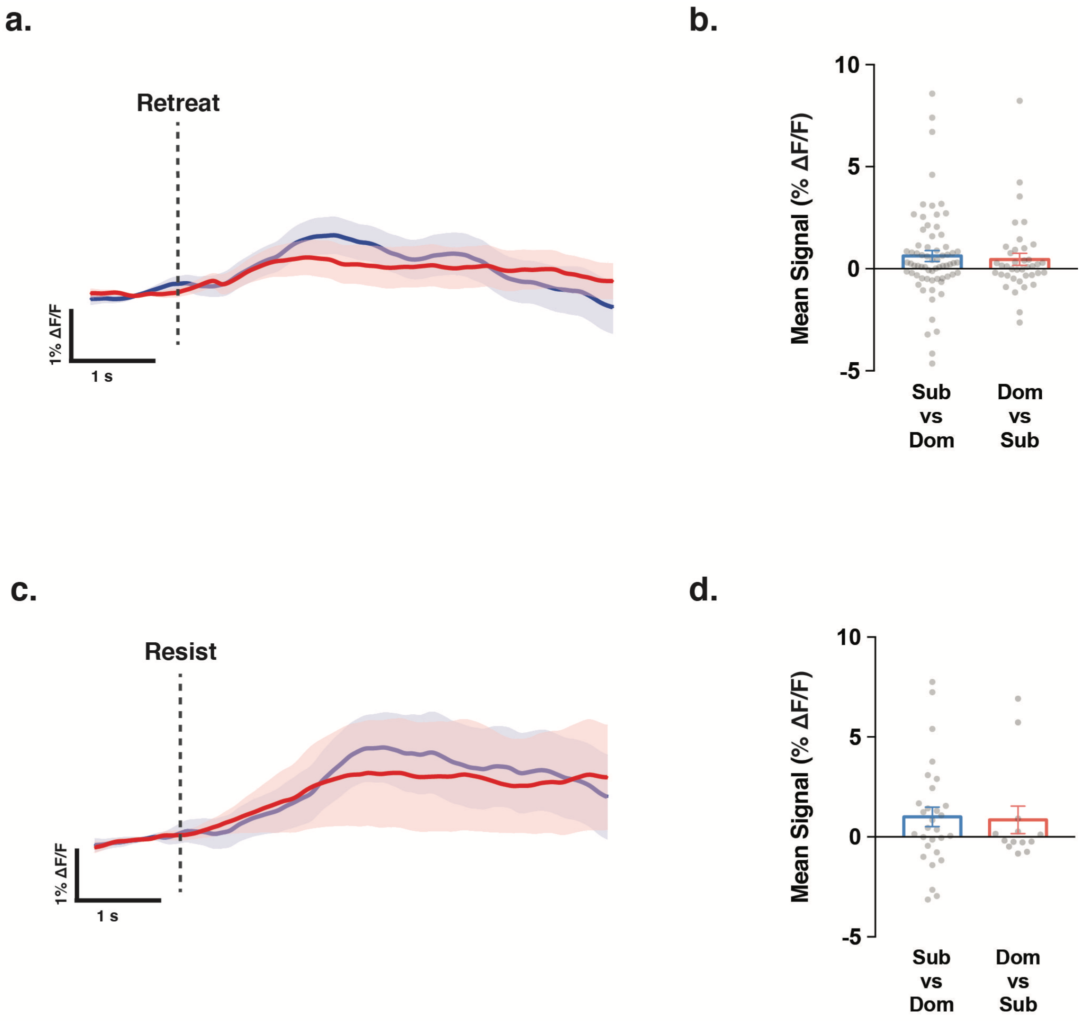

Supplementary Figure 11. Inhibiting vmPFC-NAcSh activity did not alter any tube test behaviors in subordinate or dominant animals facing novel social partners of opposing status.

a. – e. For subordinate mice expressing hM4Di or viral controls versus novel dominant mice, we found no difference in **a.** the percentage of wins ($F(1,23) = 0.446$, $p>0.05$, N = 9 hM4Di animals, 16 Control animals), **b.** the duration of bouts ($F(1,23) = 1.37$, $p>0.05$, N = 9 hM4Di animals, 16 Control animals), **c.** the rate of retreats ($F(1,23)=1.534$, $p>0.05$, N = 9 hM4Di animals, 16 Control animals), **d.** the rate of resists ($F(1,23) = 1.54$, $p>0.05$, N = 9 hM4Di animals, 16 Control animals) and the **e.** the fraction of pushes resisted ($F(1,25)=0.96$, $p>0.05$, N = 6, 7 hM4Di bouts; 10, 6 Control bouts – note: only bouts in which the partner performed a push behavior were included in this analysis) were not significantly altered with CNO injection compared to counterpart mice injected with saline. For all tests in **a. – e.** two-way repeated measures ANOVA were performed. All post-hoc comparisons were adjusted via a Holm-Bonferroni correction. Error bars presented as mean +/- SEM.

f. – k. For dominant mice expressing hM4Di or viral controls versus novel dominant mice, we found no difference in **f.** the percentage of wins ($F(1,24) = 0.806$, $p>0.05$, N = 10 hM4Di animals, 16 Control animals; 2 experiments), **g.** the duration of bouts ($F(1,24) = 0.063$, $p>0.05$, N = 10 hM4Di bouts, 16 Control bouts), **h.** the rate of pushes ($F(1,24)=2.065$, $p>0.05$, N = 10 hM4Di bouts, 16 Control bouts), **i.** the rate of retreats ($F(1,24)=0.71$, $p>0.05$, N = 10 hM4Di bouts, 16 Control bouts), **j.** the rate of resists ($F(1,24) = 1.437$, $p>0.05$, N = 10 hM4Di bouts, 16 Control bouts) and the **k.** the fraction of pushes resisted ($F(1,30)=0.75$, $p>0.05$, N = 7, 6 hM4Di bouts; 11, 10 Control bouts – note: only bouts in which the partner performed a push behavior were included in this analysis) were not significantly altered with CNO injection compared to counterpart mice injected with saline. For all tests in **a. – e.** two-way repeated measures ANOVA were performed. All post-hoc comparisons were adjusted via a Holm-Bonferroni correction. Error bars presented as mean +/- SEM.

Supplementary Figure 11 on next page

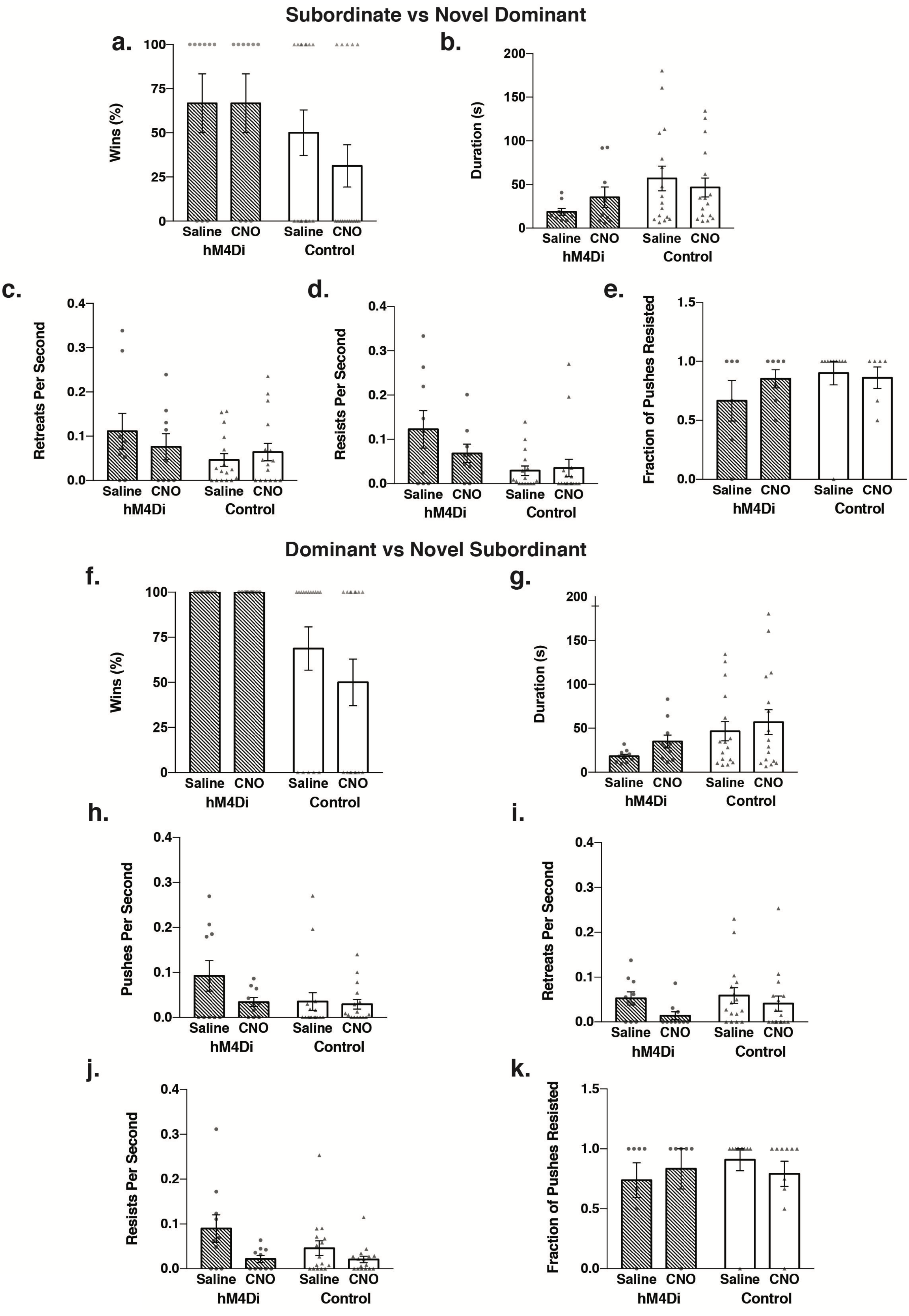
Subordinate vs Novel Dominant
a.
Wins (%)
Saline CNO Saline CNO
hM4Di Control
b.
Duration (s)
c.
Retreats Per Second
d.
Resists Per Second
e.
Fraction of Pushes Resisted
Dominant vs Novel Subordinant
f.
Wins (%)
g.
Duration (s)
h.
Pushes Per Second
i.
Retreats Per Second
j.
Resists Per Second
k.
Fraction of Pushes Resisted

Supplementary Figure 12. Additional open field social interaction behavioral quantification.

- **a.** Susceptible animals spent significantly more total time in the corner zones compared with resilient and unstressed control animals during the open field social interaction task. One way ANOVA (N = 119 animals, 8 experiments), Bonferroni corrected $F(2, 115) = 34$, ****$p < 0.0001$. Control vs susceptible $t(115) = 7.17$, ****$p < 0.0001$, control vs resilient $t(115)=0.02$ $p > 0.99$, susceptible vs resilient $t(115)=6.94$ ****$p<0.0001$. Error bars presented as mean +/- SEM.
- **b.** Control, susceptible and resilient animals do not differ in distance traveled in the open field. One way ANOVA (N = 12 control, 18 susceptible, 18 resilient animals, 2 experiments), no interaction $F(2,45) = 0.88$, $p = 0.42$. Error bars presented as mean +/- SEM.
- **c.** Control, susceptible and resilient animals do not differ in interaction zone entries during open field social interaction. One way ANOVA (N = 12 control, 18 susceptible, 18 resilient animals, 2 experiments), no interaction $F(2,45) = 2.88$, $p = 0.07$. Error bars presented as mean +/- SEM.
- **d.** To determine whether susceptibility to defeat stress as measured by social interaction behavior also generalized to other stress-sensitive behaviors, a separate cohort of N=14 mice were tested on sucrose preference and elevated plus maze assays before after chronic social defeat stress. Time spent in the open arms during the elevated plus maze task decreased for mice following CSDS. *Repeated Measures ANOVA* ($F(1,26)=139.3$, ***$p<0.0001$). However, the interaction of Group (Susceptible vs Resilient) by Condition (Naïve vs Stressed) was not significant ($F(1/26) = 0.88$, $p=0.36$), indicating comparable effects on anxiety-related behavior in both groups. Error bars presented as mean +/- SEM. Created with BioRender.com.
- **e.** Sucrose preference decreased for mice following CSDS ($F(1,26) = 14.05$, ***$p<0.0001$). However, the interaction of Group by Condition was not significant ($F(1/26)=2.70$, $p=0.11$), indicating comparable effects on anhedonia-related behavior in both groups. Error bars presented as mean +/- SEM. Created with BioRender.com.

Supplementary Figure 12 on next page

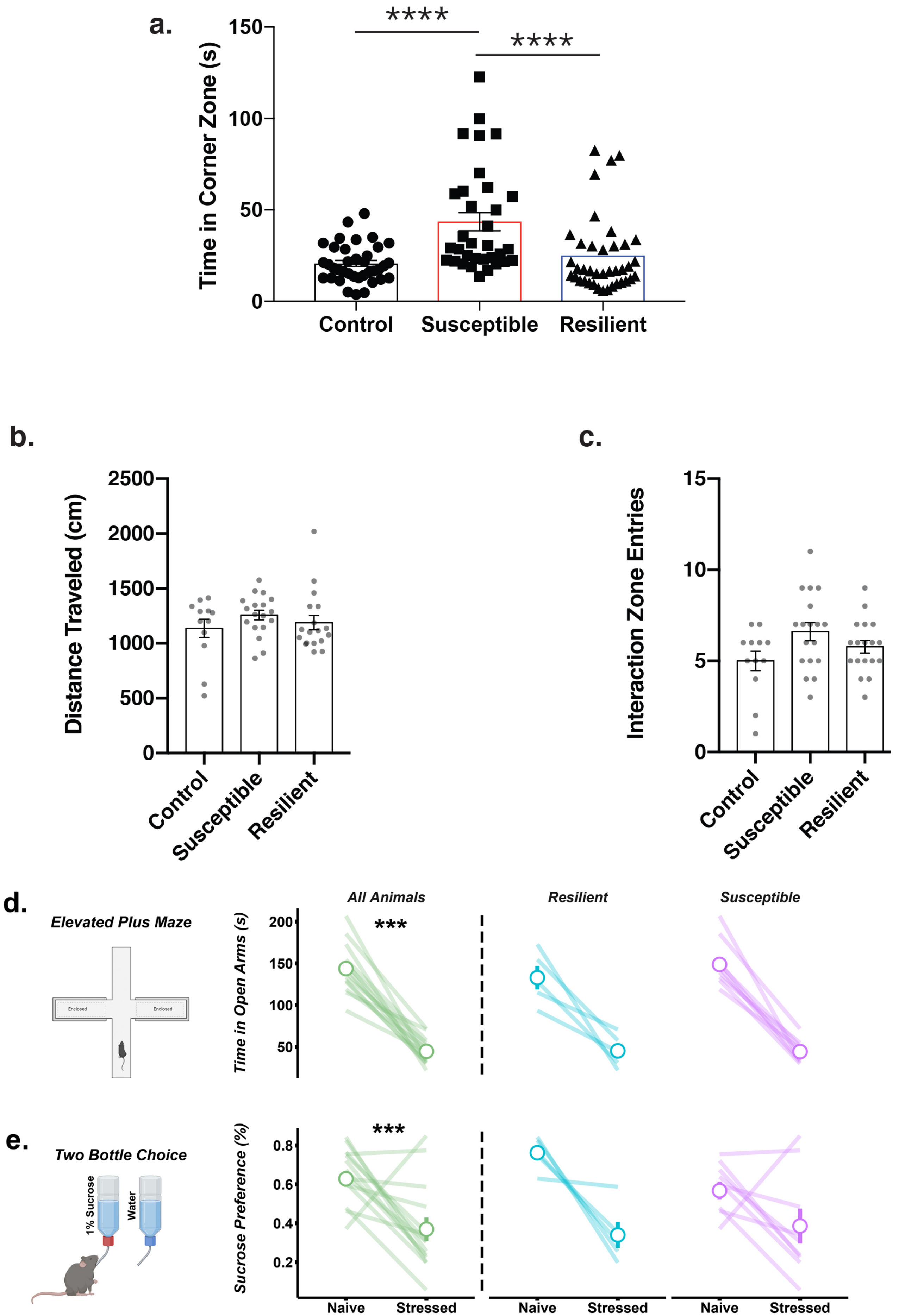
a.
Time in Corner Zone (s)
****
****
Control
Susceptible
Resilient
b.
Distance Traveled (cm)
Control
Susceptible
Resilient
c.
Interaction Zone Entries
Control
Susceptible
Resilient
d.
Elevated Plus Maze
Enclosed
Enclosed
All Animals
Resilient
Susceptible
Time in Open Arms (s)
***
e.
Two Bottle Choice
1% Sucrose
Water
Sucrose Preference (%)
***
Naive
Stressed
Naive
Stressed
Naive
Stressed

Supplementary Figure 13. Activity in vmPFC-NAcSh cells as a function of distance to the partner mouse during OFSI. In stress resilient mice, vmPFC-NAcSh activity increased upon approach and entry into the interaction zone and tended to remain elevated for the duration of the social interaction in which the test mouse and partner mouse were in close proximity. To further understand the relationship between vmPFCNAcSh activity, interaction zone entry, and proximity to the partner mouse, we plot two representative examples of vmPFC-NAcSh photometry signal (blue) from two stress resilient animals during interaction zone entries, plotted alongside the distance of the animal from the CD1 partners (orange) during open field social interaction. These plots show that vmPFC-NAcSh activity begins increasing, with some variability, around the interaction zone threshold and tends to remain elevated for most of the period in which the test mouse is located within 5–10 cm of the CD1 partner mouse.

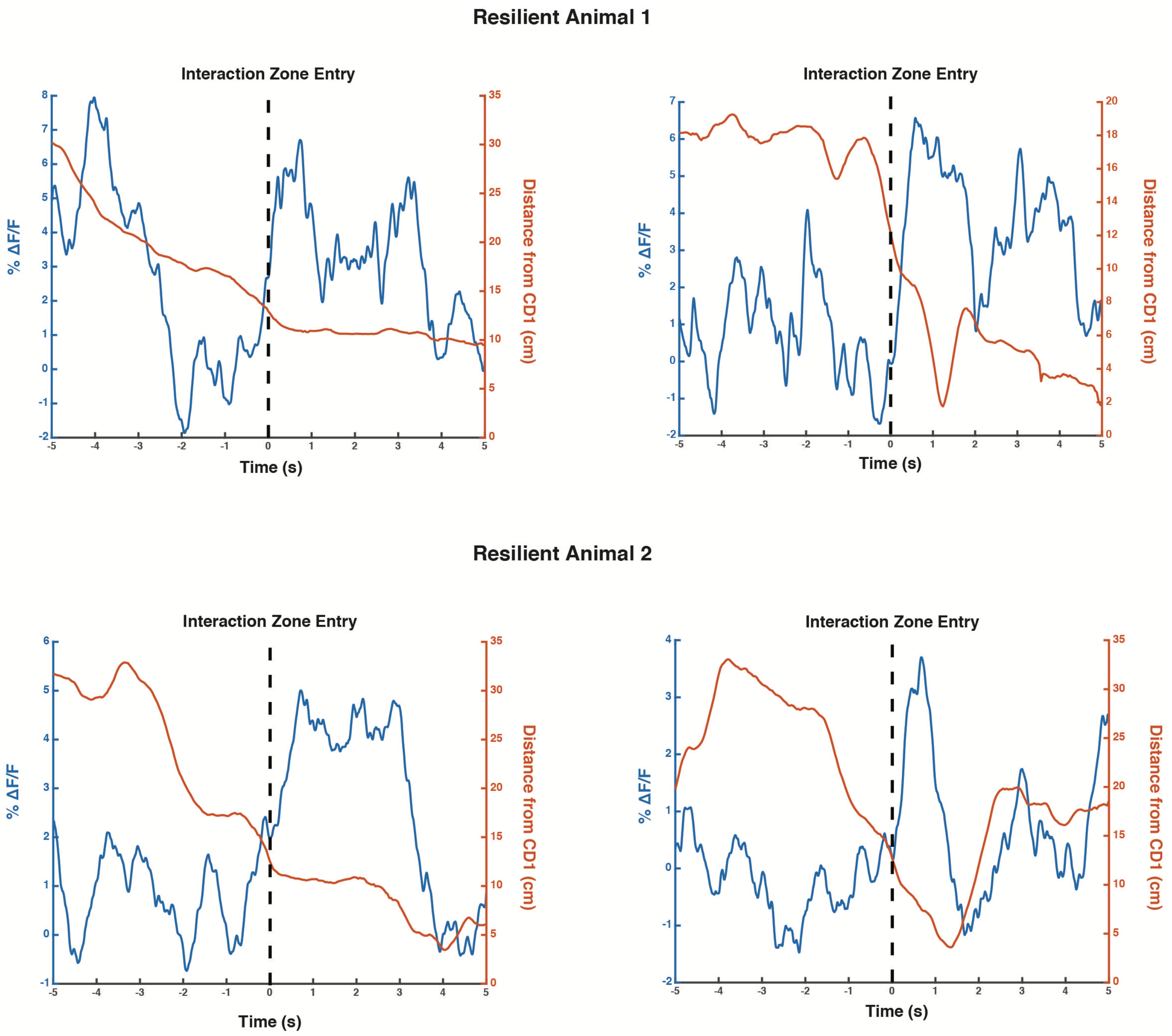

Supplementary Figure 14. vmPFC-NAcSh photometry signal levels pre and post interaction zone entry and correlation with social avoidance scores.

- **a.** There was a significant increase in the vmPFC-NAcSh photometry signal upon entering the interaction zone with a CD1 partner, average vmPFC-NAcSh circuit activity (dF/F) before (-1.5 to -0.5 s) and after (- 0.5 to 0.5 s) entry into the interaction zone, for both the stress resilient group and stress susceptible group. Linear mixed effects model, Resilient: $t(132)=5.86$, ****$p < 0.0001$. Susceptible: $t(170)=2.30$, *$p = 0.023$. Control: $t(112)=0.18$, $p = 0.86$. N = Resilient 67 events, 10 animals; Susceptible 86 events, 19 animals; Control 57 events, 13 animals, 5 experiments. Error bars presented as mean +/- SEM.
- **b.** There was no significant correlation between vmPFC-NAcSh cell activity and social avoidance score in the stress control group. Spearman $\rho = 0.15$, $p=0.62$.
- **c.** There was a significant correlation between vmPFC-NAcSh cell activity and social avoidance score across the entire stress cohort. Spearman $\rho = -0.40$, $p = 0.037$.

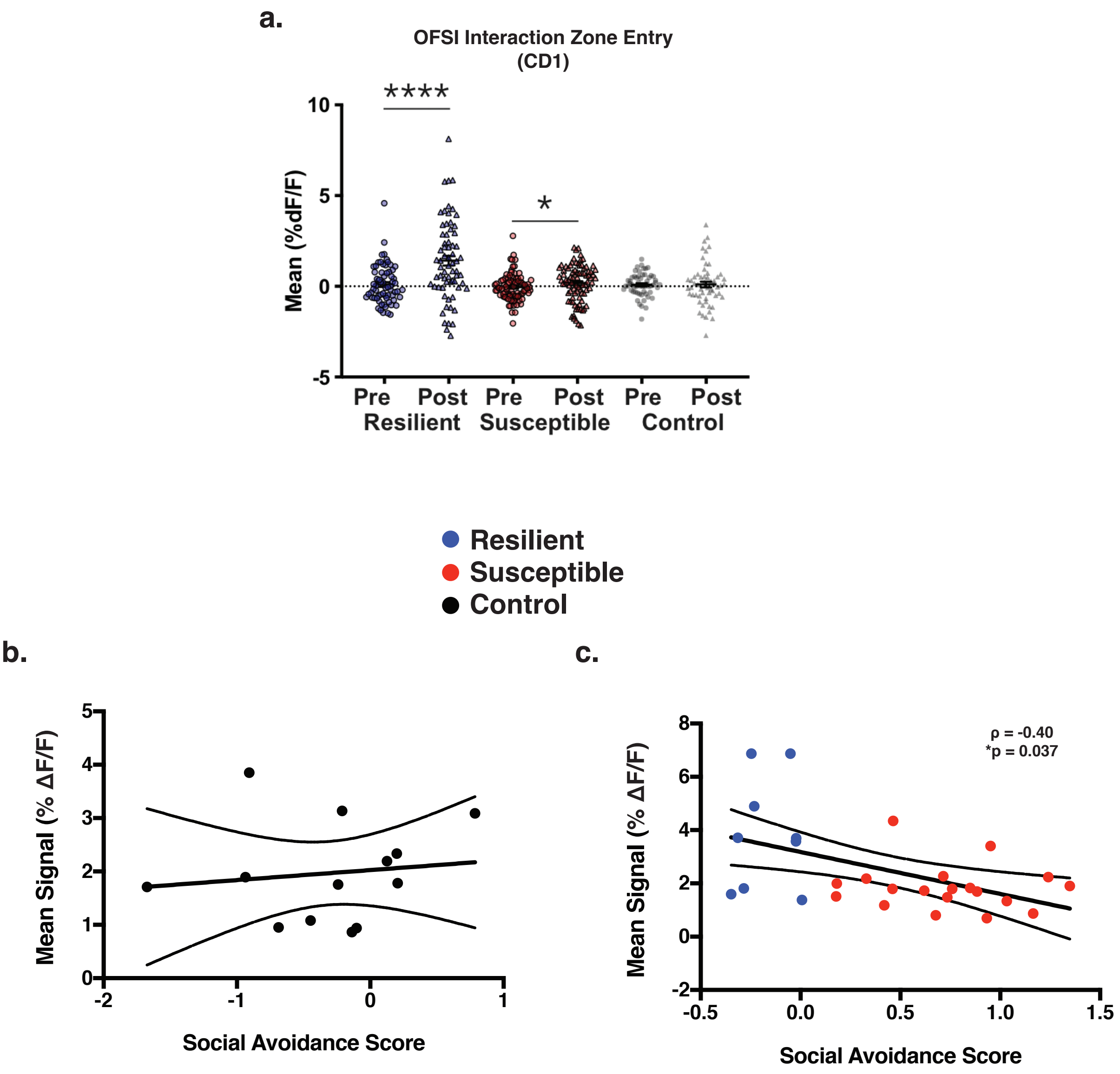

Supplementary Figure 15. vmPFC-NAcSh mean signal during interaction zone entries with unfamiliar C57 and forced interaction with CD1.

- **a.** There was no significant change in the vmPFC-NAcSh photometry signal upon entering the interaction zone with an unfamiliar C57 animal. Linear mixed effects model, Resilient: $t(36)=1.06$, $p = 0.30$. Susceptible: $t(68)=1.25$, $p = 0.22$. Control: $t(24)=1.61$, $p = 0.12$. N = Resilient 19 events, 4 animals; Susceptible 35 events, 10 animals; Control 13 events, 3 animals, 1 experiment. Error bars presented as mean +/- SEM.
- **b.** There was no significant change in the vmPFC-NAcSh photometry signal upon forced interaction with a CD1 partner. Linear mixed effects model, Resilient: $t(60)=0.51$, $p = 0.61$. Susceptible: $t(118)= -0.21$, $p = 0.83$. Control: $t(170)=-0.16$, $p = 0.87$. N = Resilient 31 events, 3 animals; Susceptible 60 events, 6 animals; Control 86 events, 8 animals, 1 experiment. Error bars presented as mean +/- SEM.

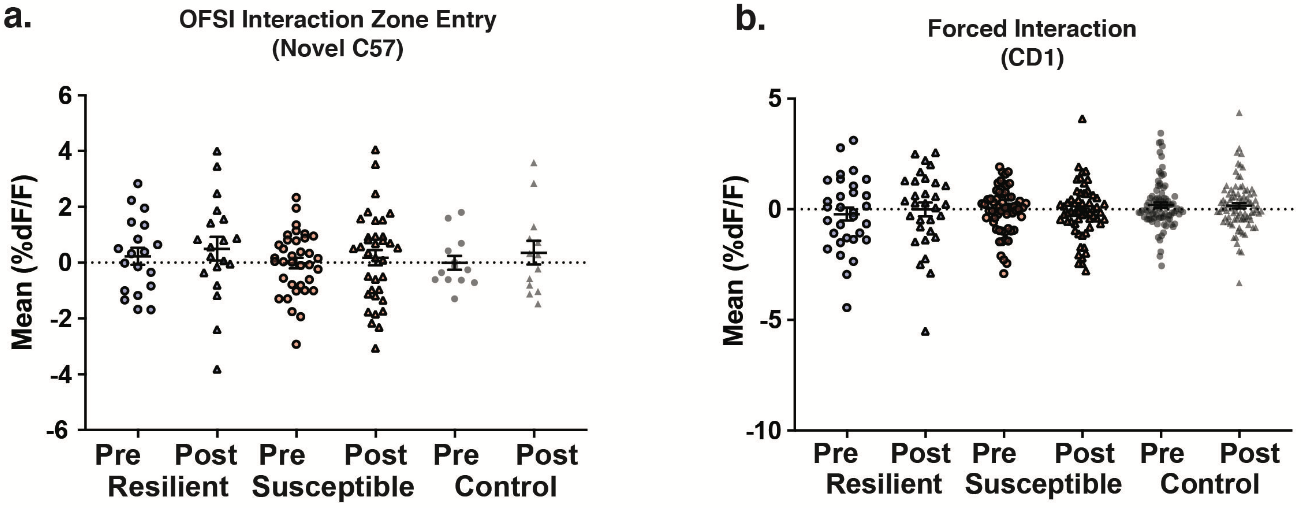

Supplementary Figure 16. Social avoidance scores of stress groups are stable with repeated open field social interaction testing.

a. Social avoidance score pairs for each animal on Day 1 and Day 2 of testing for each stress phenotype group. Repeated measures two way ANOVA (N = 11 control, 15 susceptible, 15 resilient), no interaction $F(2,38)=0.2686$, $p=0.77$. Error bars presented as mean +/- SEM.

b. Social avoidance scores on Day 1 of open field social interaction test were significantly correlated with social avoidance scores on Day 2 of open field social interaction test. N = 40; Spearman $\rho = 0.46$; $**p = 0.0025$.

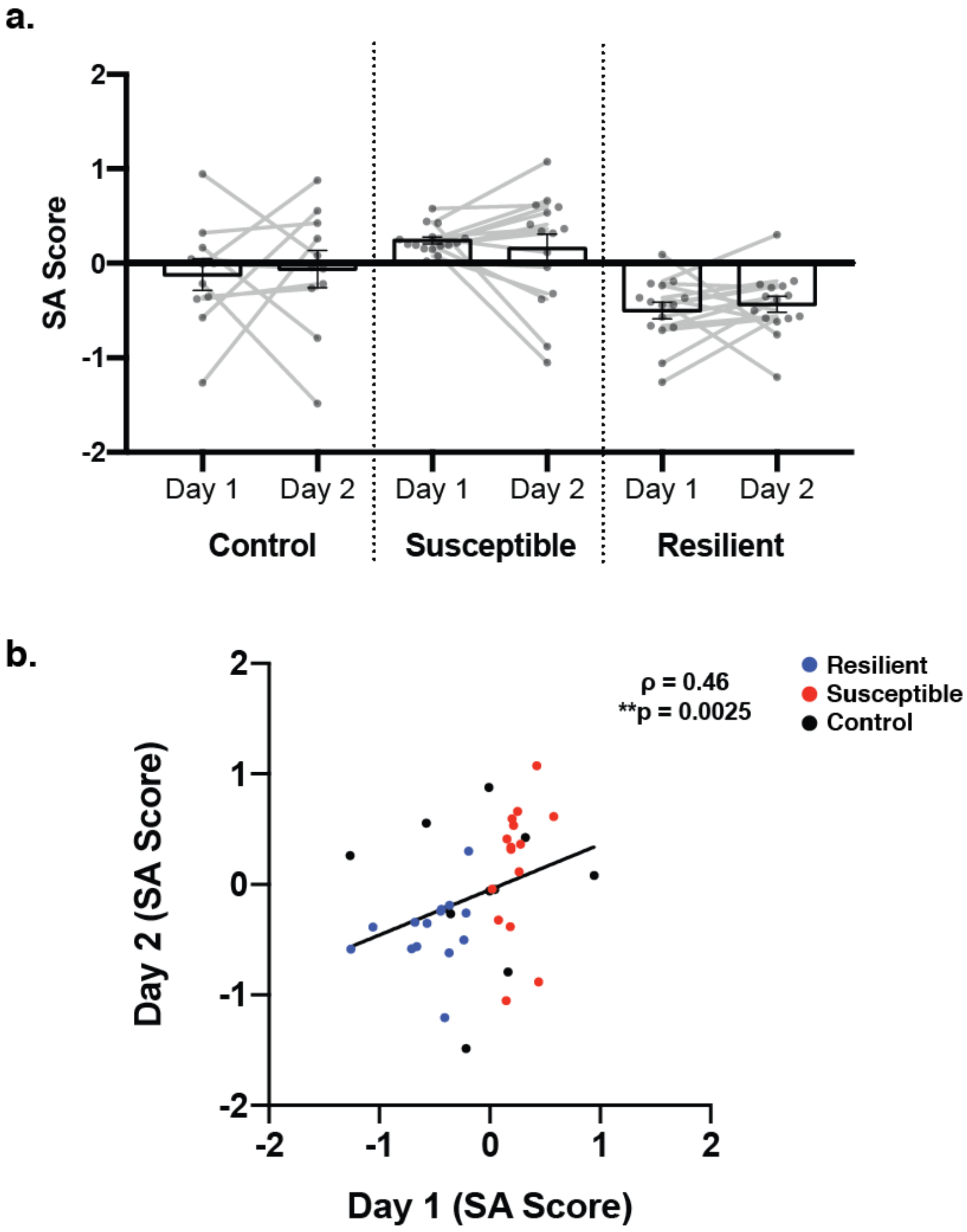

Supplementary Figure 17. Saline controls for vmPFC-NAcSh inhibition during social approach behavior following social defeat.

- **a.** Experimental timeline and schematic of surgical strategy for saline control animals which were virally injected with either hM4DI-mCherry inhibitory DREADS or viral controls in vmPFC-NAcSh cells. Created with BioRender.com
- **b.** Social interaction behavior (total interaction zone time) of both stressed (susceptible (red) and resilient (blue)) and unstressed hM4Di expressing animals was not changed by saline injection compared to viral control animals. Repeated measures two way ANOVA (N = Unstressed: 4 viral control, 4 hM4Di; Stressed: 17 viral control, 18 hM4Di), non-significant interaction $F(1,39)=9.086$, $p=0.9248$.

a.

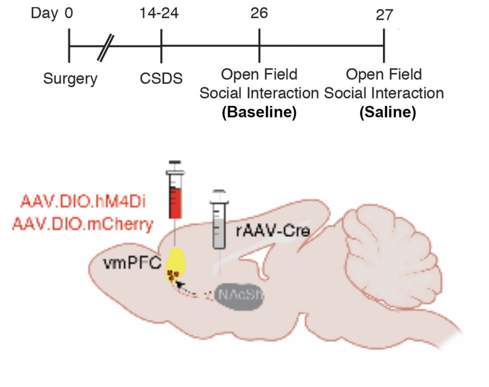

b.

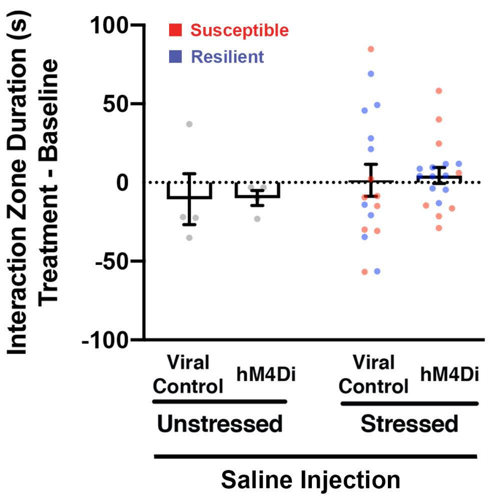

Supplementary Figure 18. Social interaction behavior changes in unstressed, susceptible and resilient animals during vmPFC-NAcSh inhibition. Two way ANOVA (Virus x Condition) (N = 5 viral control injected unstressed control animals, 11 hM4Di injected unstressed control animals, 9 viral control injected susceptible animals, 9 hM4Di injected susceptible animals, 8 viral control injected resilient animals, 6 hM4Di injected resilient animals), Bonferroni corrected for post-hoc comparisons, significant interaction $F(2,42) = 3.3$, $^*p = 0.04$; resilient viral control vs. resilient hM4Di: $t(42) = 1.93$, $p = 0.18$; susceptible viral control vs. susceptible hM4Di: $t(42) = 1.95$, $p = 0.17$; unstressed viral control vs. unstressed hM4Di: $t(42) = 1.3$, $p = 0.6$. Error bars presented as mean +/- SEM.

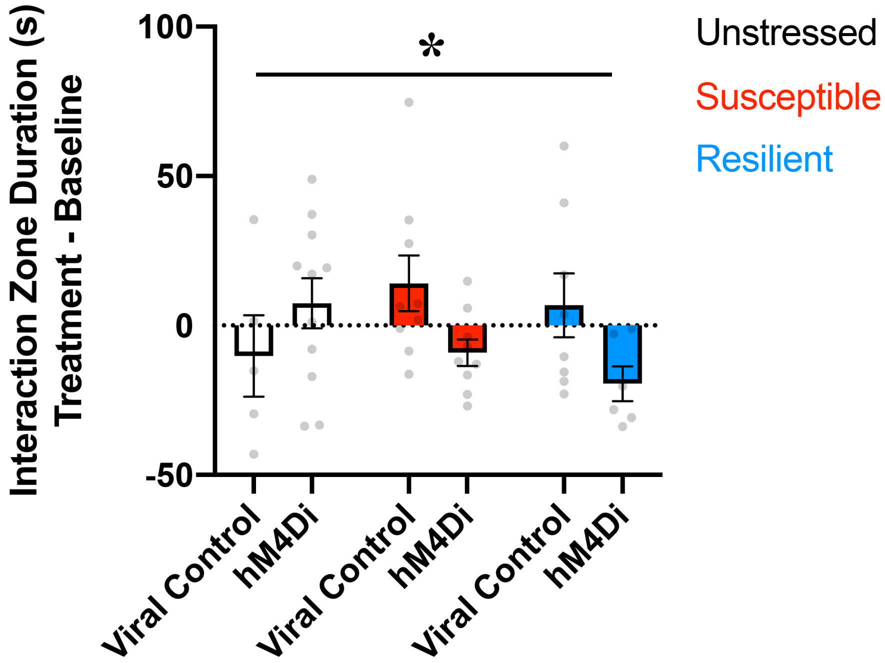

Supplement: Supplementary file 1 — Supplementary Information [file 41467_2023_37460_MOESM1_ESM.pdf]
