# Supplementary material for: Regulation of social interaction in mice by a frontostriatal circuit modulated by established hierarchical relationships
Source: Nat Commun. 2023 Apr 29;14:2487. doi: 10.1038/s41467-023-37460-6 (PMC10148889; doi:10.1038/s41467-023-37460-6)
Supplement: Supplementary file 2 — Description of Additional Supplementary Files [file 41467_2023_37460_MOESM2_ESM.pdf]

Description of Additional Supplementary Files

File Name: Supplementary Movie 1
Description: Example tube test bout to demonstrate different push rates. Left animal push rate is 0.12 pushes per second; right animal push rate is 0.078 pushes per second.

File Name: Supplementary Movie 2
Description: Example tube test bout to demonstrate different push rates. Left animal push rate is 0.039 pushes per second; right animal push rate is 0.039 pushes per second.

File Name: Supplementary Movie 3
Description: Example tube test bout to demonstrate different push rates. Left animal push rate is 0.064 pushes per second; right animal push rate is 0.016 pushes per second.
