# Supplementary material for: Regulation of social interaction in mice by a frontostriatal circuit modulated by established hierarchical relationships
Source: Nat Commun. 2023 Apr 29;14:2487. doi: 10.1038/s41467-023-37460-6 (PMC10148889; doi:10.1038/s41467-023-37460-6)
Supplement: Supplementary file 6 — Reporting Summary [file 41467_2023_37460_MOESM6_ESM.pdf]

Corresponding author(s): Conor Liston, Anjali Rajadhyaksha

Last updated by author(s): Sep 29, 2022

Reporting Summary

Nature Portfolio wishes to improve the reproducibility of the work that we publish. This form provides structure for consistency and transparency in reporting. For further information on Nature Portfolio policies, see our Editorial Policies and the Editorial Policy Checklist.

Statistics

For all statistical analyses, confirm that the following items are present in the figure legend, table legend, main text, or Methods section.

n/a	Confirmed	
☐	☒	The exact sample size (n) for each experimental group/condition, given as a discrete number and unit of measurement
☐	☒	A statement on whether measurements were taken from distinct samples or whether the same sample was measured repeatedly
☐	☒	The statistical test(s) used AND whether they are one- or two-sided<br>*Only common tests should be described solely by name; describe more complex techniques in the Methods section.*
☐	☒	A description of all covariates tested
☐	☒	A description of any assumptions or corrections, such as tests of normality and adjustment for multiple comparisons
☐	☒	A full description of the statistical parameters including central tendency (e.g. means) or other basic estimates (e.g. regression coefficient) AND variation (e.g. standard deviation) or associated estimates of uncertainty (e.g. confidence intervals)
☐	☒	For null hypothesis testing, the test statistic (e.g. F, t, r) with confidence intervals, effect sizes, degrees of freedom and P value noted<br>*Give P values as exact values whenever suitable.*
☒	☐	For Bayesian analysis, information on the choice of priors and Markov chain Monte Carlo settings
☐	☒	For hierarchical and complex designs, identification of the appropriate level for tests and full reporting of outcomes
☐	☒	Estimates of effect sizes (e.g. Cohen's d, Pearson's r), indicating how they were calculated

Our web collection on statistics for biologists contains articles on many of the points above.

Software and code

Policy information about availability of computer code

Data collection	Behavioral tracking was colledted using Ethovision 11.5 XT, Noldus, Information Technology; Leica Application Suite Advanced Fluorescence 3.0.0 build 8134 257 software, Fiber photometry was performed as previously described (Gunaydin et al., 2014) via a custom built rig.
Data analysis	Automated scoring utilized DeepLabCut (http://ww.mousemotorlab.org/deeplabcut); Hand scoring was facilitated by the CowLog program (http://cowlog.org/); Fiber photometry analyses were based upon previously described work (http://clarityresourcecenter.com/fiberphotometry.html); Prism 7.0d (GraphPad software) or MATLAB (Mathworks) were utilized for plotting and statistical anlayses.

For manuscripts utilizing custom algorithms or software that are central to the research but not yet described in published literature, software must be made available to editors and reviewers. We strongly encourage code deposition in a community repository (e.g. GitHub). See the Nature Portfolio guidelines for submitting code & software for further information.

Data

Policy information about availability of data

All manuscripts must include a data availability statement. This statement should provide the following information, where applicable:

- Accession codes, unique identifiers, or web links for publicly available datasets
- A description of any restrictions on data availability
- For clinical datasets or third party data, please ensure that the statement adheres to our policy

The main data supporting the results in this study are provided with this paper (please see attached source data). Pre-processed data is available from the corresponding authors within reasonable request.

Human research participants

Policy information about studies involving human research participants and Sex and Gender in Research.

Reporting on sex and gender	No human research participants were used in this study
Population characteristics	No human research participants were used in this study
Recruitment	No human research participants were used in this study
Ethics oversight	No human research participants were used in this study

Note that full information on the approval of the study protocol must also be provided in the manuscript.

Field-specific reporting

Please select the one below that is the best fit for your research. If you are not sure, read the appropriate sections before making your selection.

☒ Life sciences ☐ Behavioural & social sciences ☐ Ecological, evolutionary & environmental sciences

For a reference copy of the document with all sections, see nature.com/documents/nr-reporting-summary-flat.pdf

Life sciences study design

All studies must disclose on these points even when the disclosure is negative.

Sample size	For fiber photometry experiments, sample size was estimated using prior studies in the field (ie: Gunaydin et al., 2014; Lerner et al., 2015). For tube-test behavioral experiments, sample size was estimated using prior studies in the field (ie: Zhou et al., 2017;).
Data exclusions	Exclusion criteria were established prior to experimentation. For fiber photometry experiments, exclusion criteria consisted of animals showing poor signal as defined by a <1% signal difference between the 95th and 5th percentile of signal. For all experiments, outliers were removed using the Grubb test where alpha was set to <0.05.
Replication	Replicates of experiments are noted in methods ant text. All replicates that were performed were successful. Replication varied across experiments, however on average experiments were performed once.
Randomization	Mice were randomly distributed into control and stress groups for all stress studies. For within cage hierarchical studies groupings were determined by behavioral results as described in methods.
Blinding	Behavioral scoring was run via an automated tracking set up, investigator running the program was blinded to animal groups.

Reporting for specific materials, systems and methods

We require information from authors about some types of materials, experimental systems and methods used in many studies. Here, indicate whether each material, system or method listed is relevant to your study. If you are not sure if a list item applies to your research, read the appropriate section before selecting a response.

Materials & experimental systems

n/a	Involved in the study
☐	☒ Antibodies
☒	☐ Eukaryotic cell lines
☒	☐ Palaeontology and archaeology
☐	☒ Animals and other organisms
☒	☐ Clinical data
☒	☐ Dual use research of concern

Methods

n/a	Involved in the study
☒	☐ ChIP-seq
☒	☐ Flow cytometry
☒	☐ MRI-based neuroimaging

Antibodies

Antibodies used

Chicken Anti-GFP (Abcam, ab13970), Goat Anti-Chicken Alexa 488 (ThermoFisher, #A-11039), Rabbit Anti-mCherry (Abcam, ab167453), Goat anti-rabbit (Abcam, ab150077). Primary antibodies were diluted 1:200. Secondary antibodies were diluted 1:1000.

Validation

Validation of antibodies were performed by respective manufacturers . As described on the Abcam website:
"Antibody specificity is confirmed by looking at cells that either do or do not express the target protein within the same tissue. Initially, our scientists (Abcam) will review the available literature to determine the best cell lines and tissues to use for validation. We then check the protein expression by IHC/ICC to see if it has the expected cellular localization. If the localization of the signal is as expected, this antibody will pass and is considered suitable for use in IHC/ICC."

ThermoFisher describes their validation process as:

"Invitrogen antibodies are currently undergoing a rigorous two-part testing approach
Part 1—Target specificity verification
This helps ensure the antibody will bind to the correct target. Our antibodies are being tested using at least one of the following methods to ensure proper functionality in researcher’s experiments. Click on each testing method below for detailed testing strategies, workflow examples, and data figure legends.

Knockout—expression testing using CRISPR-Cas9 cell models
Knockdown—expression testing using RNAi to knockdown gene of interest
Independent antibody verification (IAV)—measurement of target expression is performed using two differentially raised antibodies recognizing the same protein target
Cell treatment—detecting downstream events following cell treatment
Relative expression—using naturally occurring variable expression to confirm specificity
Neutralization—functional blocking of protein activity by antibody binding
Peptide array—using arrays to test reactivity against known protein modifications
SNAP-ChIP™—using SNAP-ChIP to test reactivity against known protein modifications
Immunoprecipitation-Mass Spectrometry (IP-MS)—testing using immunoprecipitation followed by mass spectrometry to identify antibody targets
Part 2—Functional application validation
These tests help ensure the antibody works in a particular application(s) of interest, which may include
(but are not limited to):

Western blotting
Flow cytometry
ChIP
Immunofluorescence imaging
Immunohistochemistry
Most antibodies were developed with specific applications in mind. Testing that an antibody generates acceptable results in a specific application is the second part of confirming antibody performance."

Data sheets and example images are available on the website of manufacturers.

Animals and other research organisms

Policy information about studies involving animals; ARRIVE guidelines recommended for reporting animal research, and Sex and Gender in Research

Laboratory animals	Adults (~postnatal day 60-70) male wildtype C57BL/6J (Jackson Laboratories) and retired male CD1 breeders aged 4-6 months (Charles River Laboratories)
Wild animals	No wild animals were used in this study
Reporting on sex	Findings apply to male sex. Only male mice were used in this study.
Field-collected samples	No field-collected samples were used in this study
Ethics oversight	Weill Cornell Medicine Institutional Animal Care and Use Committee

Note that full information on the approval of the study protocol must also be provided in the manuscript.
